# Surface carbonation governs olivine reactivity for rapid CO_2_ mineralization

**DOI:** 10.1038/s41467-026-78548-z

**Published:** 2026-10-14

**Authors:** Zihua Shao, Yunfeng Liang, Gyuhwan Jo, Keisuke Nakamura, Takeshi Tsuji

**Affiliations:** 1 Department of Systems Innovation, School of Engineering, The University of Tokyo, Tokyo, Japan; 2 e-Technology Innovation Center, ENEOS Xplora Inc., Tokyo, Japan

**Keywords:** Carbon capture and storage, Geochemistry, Reaction mechanisms, Reaction kinetics and dynamics, Density functional theory

## Abstract

Ultramafic rocks are a global resource with vast potential for permanent CO_2_ mineralization. However, the origin of the striking reactivity difference between Ca- and Mg-bearing olivine remains poorly understood. Here we show that this difference arises not merely from the type of cation, but more fundamentally from how these cations modulate the oxygen reactivity. Nonbridging oxygens serve as the oxygen source for CO_2_ surface reactions, which in Ca-olivine reduce the Ca^2+^ dissolution barrier by 60% and accelerate ion release by six orders of magnitude. The calculated apparent dissolution rate constants reproduce the experimentally observed complete conversion of Ca-olivine within hours under 1 atm CO_2_. This multiscale framework reveals how surface reactions govern silicate carbonation and provides a predictive basis for modeling and engineering large-scale CO_2_ mineralization in both synthetic materials and basaltic reservoirs.

## Abstract (web summary)

The authors explain the role of reactivity contrast between Ca^2+^ and Mg^2+^ in olivine group silicates for CO_2_ removal and show that surface reactions with CO_2_ accelerate mineral dissolution, helping elucidate rapid, permanent carbon storage in rocks.

## Introduction

Achieving net-zero emissions has intensified interest in scalable and durable carbon dioxide removal (CDR) strategies. Ultramafic rock (such as olivine) serves as a key reactive mineral for long-term CDR solutions by mineral carbonation, underpinning strategies such as CO_2_ removal materials, enhanced weathering, and in situ carbon mineralization. Ca-olivine (Ca_2_SiO_4_), synthesized via Ca^2+^/Mg^2+^ exchange reactions, offers a highly reactive and energy-efficient alternative for CO_2_ removal, enabling rapid carbonation under ambient conditions^1^. Global reserves of Mg-rich silicates have the potential to remove 10^5^−10^8^ gigatons (Gt) CO_2_^2^ and annual production of mine tailings about 420 million tons^3^, this material offers vast and scalable potential for permanent CDR. Basaltic rocks, composed primarily of olivine, pyroxene, and plagioclase, are globally abundant, with olivine being the most reactive phase during enhanced weathering^4^ and exhibiting the fastest cation release rates under acidic and neutral conditions relevant to in-situ carbon mineralization^5^. Applying crushed basalt to croplands as an enhanced weathering approach could help achieve global CDR targets of 0.5–2 Gt CO_2_ yr^−1^, with China, India, the USA, and Brazil identified as key regions due to their large agricultural areas and basalt availability^6^. Olivine weathers the fastest among major silicates and volcanic glasses, the deployment of which indicates a carbon sequestration potential of 3.7–18.4 Gt CO_2_ per year^7^. In subsurface conditions, carbon mineralization strategies inject CO_2_-rich fluids into basaltic reservoirs rich in olivine, leveraging similar carbonation mechanisms^8^. In situ carbonation of peridotite, an ultramafic rock dominated by olivine, could consume over 1 Gt CO_2_ per year in Oman alone^9^. 88% of the injected CO_2_ in peridotite was converted to carbonate mineral within 45 days^10^. Based on the global reserves of mafic and ultramafic rocks, carbon mineralization has a theoretical potential of sequestrating up to 6 × 10^7^ Gt CO_2_ if all resources are fully carbonated^2^. This capacity vastly exceeds the remaining global budget of 235 Gt CO_2_ for limiting global warming to 1.5 °C^11^.

Despite these promising strategies, the carbonation rate varies significantly depending on mineral composition and reaction pathway. Carbonation in basalt proceeds much more slowly than in CDR materials (Ca_2_SiO_4_) due to the reactivity contrast between Ca and Mg in olivine; natural basalts are Mg-rich and contain less than 1% Ca^12^. The prevailing mechanism for mineral carbonation involves a dissolution–precipitation process in aqueous environments^13^, where CO_2_ dissolves to form HCO_3_^−^, creating a mildly acidic condition that facilitates the release of Ca^2+^ and Mg^2+^. These cations then react with dissolved carbonate to precipitate as stable carbonates^14^. Despite extensive efforts, this aqueous-phase pathway alone cannot explain the rapid mineralization observed in field-scale projects, such as CarbFix and Wallula, where over 60% of injected CO_2_ was mineralized within two years^8,15^. In the absence of direct CO_2_-rock interactions, carbonation requires over a century to complete^16^. This discrepancy suggests that additional carbonation pathways, particularly direct CO_2_–mineral surface reactions and surface complexation processes, may contribute to mineralization but are not explicitly represented in conventional dissolution–precipitation-based models. Direct gas–solid surface reactions are represented by reaction (1) on the mineral surface^14,17^:

1 $${{{{\rm{Ca}}}}/{{{\rm{Mg}}}}-{{{\rm{silicate}}}}}_{({{{\rm{s}}}})}\,+{{{{\rm{CO}}}}}_{2({{{\rm{g}}}})}\to {({{{\rm{Ca}}}}/{{{\rm{Mg}}}}){{{\rm{CO}}}}}_{3({{{\rm{s}}}})}+{{{{\rm{SiO}}}}}_{2({{{\rm{s}}}})}$$

Although long considered kinetically negligible^14^, here we propose that these surface reactions proceed with low activation barriers and can initiate carbonation processes that complement or even precede aqueous-phase pathways.

Previous studies have investigated olivine carbonation from various angles. Ab initio molecular dynamics simulations have been employed to investigate water and CO_2_ adsorption on five types of olivine (Mg, Ca, Mn, Fe, Co), revealing that Ca-olivine exhibits the most exothermic adsorption energies for both species^18^. Dissociative H_2_O adsorption occurs only on the Ca-olivine surface, indicating its distinct surface reactivity^19^. Classical molecular dynamics simulations have explored the role of interfacial water in modulating CO_2_ interactions at forsterite (the Mg-end member of olivine) surfaces in contact with supercritical CO_2_/H_2_O mixtures^20^. The dissolution rates of olivine have been shown to correlate with the ligand exchange kinetics of their constituent divalent cations, reflecting the influence of cation-oxygen bond character on mineral reactivity^21^. Metadynamics simulations have compared the dissolution behavior of Ca^2+^ and Mg^2+^ from olivine, revealing a significantly lower energy barrier for Ca^2+^ release^22^. More broadly, pathway-based free-energy methods such as metadynamics have been shown to provide mechanistic insight into ion detachment from solid–water interfaces^23^. Geochemical modeling has examined the influence of petrophysical parameters on mineralization rates in aqueous systems, identifying forsterite as a dominant contributor to carbonate precipitation^24^. However, these studies primarily focused on bulk dissolution–precipitation and largely neglected the role of surface complexation reactions. In this work, we present a unified, multiscale framework that links atomistic-scale surface speciation to macroscopic carbonation kinetics, following the spirit of Blum and Lasaga^25^. By integrating ab initio molecular dynamics (AIMD), Metadynamics (MTD), and geochemical modeling, we systematically investigate surface reactions, metal ion dissolution, and carbonate precipitation pathways. This framework captures how CO_2_ interacts with surface metal sites, alters local coordination environments, and drives mineral transformation through either direct carbonation or dissolution-precipitation mechanisms. The framework reveals how surface nonbridging oxygens and metal sites collectively modulate reactivity, offering theoretical guidance for the design of engineered CDR materials, enhanced weathering strategies, and in situ carbon mineralization.

## Results

### Surface reactivity of olivine with CO_2_

**Fig. 1:**
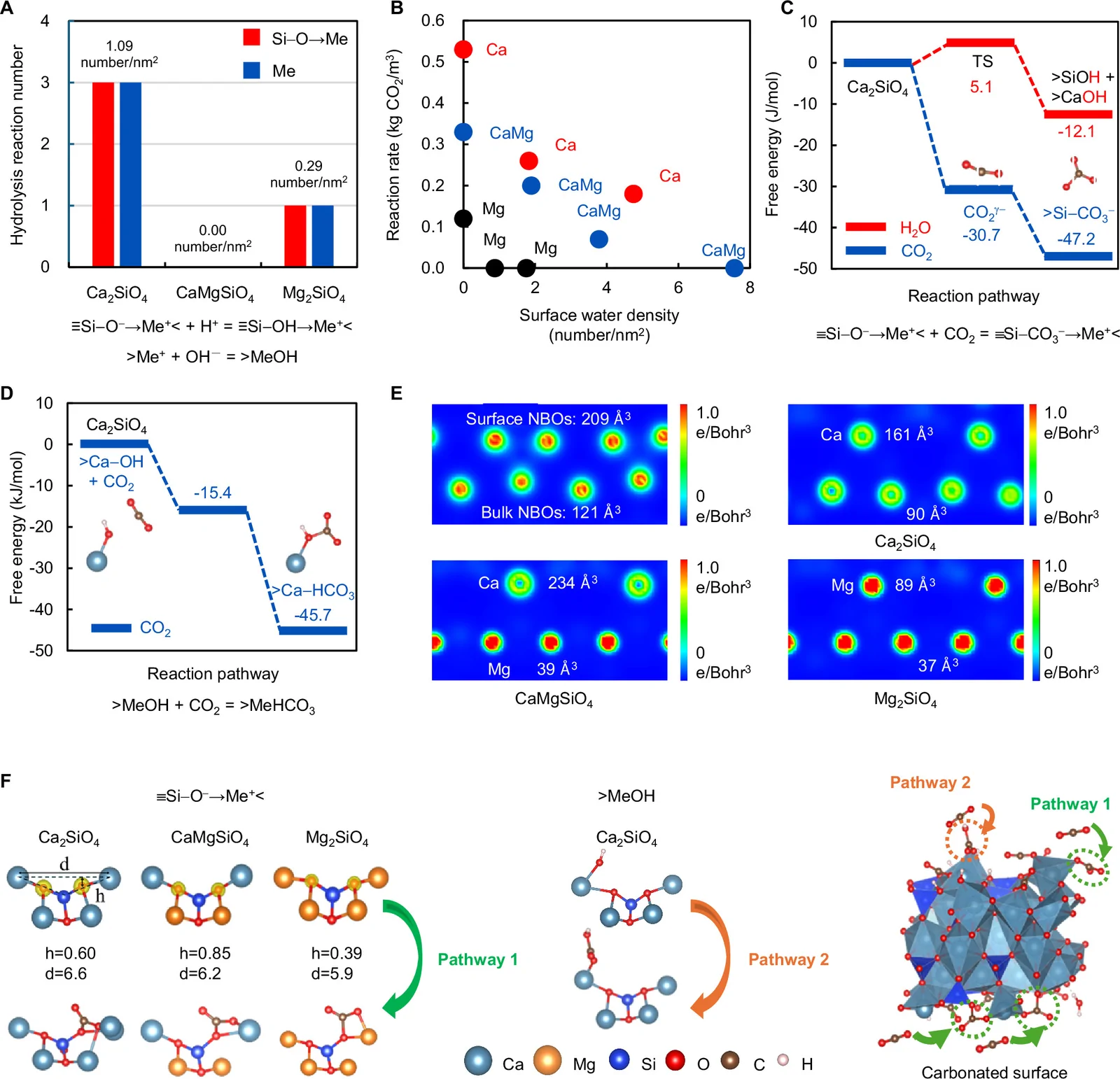
Drastic reactivity difference between Ca and Mg in olivine. **A** Hydrolysis reactions on olivine surfaces and reacted water number per surface area. **B** CO_2_ surface reaction rates under varying water coverage. **C** Binding energies of H_2_O and CO_2_ (pathway 1) on Ca_2_SiO_4_ surfaces. **D** Binding energy of CO_2_ with Ca-OH groups (pathway 2). **E** Electron density distribution and atomic volumes of NBOs in Ca-olivine, Ca in Ca-olivine, Ca/Mg in CaMg-olivine, and Mg in Mg-olivine, respectively. **F** Schematic illustration of CO_2_ surface structure on Ca-, CaMg-, and Mg-olivine before and after reaction. h and d in the unit of Å. The figure shows that the surface nonbridging oxygen (NBO) reacts more easily with CO_2_ compared with water, and the surface reactivity with CO_2_ follows an order: Ca-olivine > CaMg-olivine > Mg-olivine, stemmed from different ionic radius and surface geometries.

We have studied three different olivine mineral materials with analogous crystal structures (Ca_2_SiO_4_, CaMgSiO_4_, and Mg_2_SiO_4_). Ca-olivine (Ca_2_SiO_4_) exhibits exceptional reactivity for CDR not solely due to its Ca^2+^ content for carbonate precipitation, but because its surfaces are inherently rich in nonbridging oxygens (NBOs), highly reactive sites for both hydrolysis and carbonation. As shown in Fig. 1a, AIMD simulations reveal that freshly cleaved Ca_2_SiO_4_ surfaces undergo rapid hydrolysis, where NBOs are protonated to form Si−OH → Ca configurations and surface Ca^2+^ coordinate with OH^−^ to form Ca−OH groups. In contrast, CaMgSiO_4_ and Mg_2_SiO_4_ display reduced reactivity, as Mg^2+^ coordination stabilizes adjacent NBOs and inhibits their protonation. The limited reactivity of Mg_2_SiO_4_ surfaces aligns with prior observations that dissociated water molecules spontaneously recombine, implying that associative rather than dissociative adsorption is energetically preferred on these surfaces^19,20^. Notably, even after extended hydrolysis, a significant fraction of surface NBOs remains accessible on all surfaces, indicating their potential role as initiation sites for CO_2_ activation.

To evaluate CO_2_ surface reactivity, AIMD simulations were performed by systematically replacing surface water molecules with CO_2_ under varying surface water densities (Fig. 1b). Ca_2_SiO_4_ consistently exhibited the highest reactivity, followed by CaMgSiO_4_ and Mg_2_SiO_4_, in agreement with experimental carbonation efficiencies after 25 days: 72% for Ca_2_SiO_4_, 33% for Ca_3_Mg(SiO_4_)_2_, 21% for CaMgSiO_4_, and negligible reactivity for Mg_2_SiO_4_^1^. Two distinct surface reaction pathways for CO_2_ uptake were identified: (1) CO_2_ reacts with NBOs to form surface carbonate (CO_3_^2–^) species, and (2) reaction with metal-coordinated hydroxyls (Me–OH) to form surface bicarbonate (HCO_3_^–^) species. While both pathways are active on Ca_2_SiO_4_, the second is exclusive on this surface due to its propensity to form Ca–OH groups via hydrolysis, enabled by the strong reactivity of Ca-coordinated NBOs with water. Thermodynamics analysis shows that pathway 2 has a higher equilibrium constant (K) than pathway 1, underscoring the critical role of hydrolysis in promoting surface carbonation (Supplementary Note 1). Structural analysis further reveals the origin of this selectivity. In CaMgSiO_4_, surface NBOs coordinate to a surface Ca^2+^ and an inner Mg^2+^, whereas in Ca_2_SiO_4_ they are coordinated to two Ca^2+^ ions, and in Mg_2_SiO_4_ to two Mg^2+^ ions. The presence of Mg^2+^, with its smaller radius and stronger electrostatic field, leads to electronic stabilization of NBOs and reduces their nucleophilicity. As a result, the CO_2_ reactivity of CaMgSiO_4_ and Mg_2_SiO_4_ is suppressed relative to Ca_2_SiO_4_.

Nudged elastic band (NEB) calculations (Fig. 1c–d) reveal that CO_2_ reactions on Ca_2_SiO_4_ surface via both pathways are barrierless and exothermic, whereas the hydrolysis reaction has a barrier of 5 kJ/mol. These results indicate that NBOs preferentially react with CO_2_ over H_2_O. For comparison, the homogeneous reaction OH^−^ + CO_2_ → HCO_3_^−^ in water has a free energy barrier of 8-13 kcal/mol^26^. Therefore, the reaction between CO_2_ and the OH^−^ ligand of surface Ca^2+^ has no barrier and is spontaneous compared to its reaction with OH^−^ in water. The observation of such reactions within 10 ps in AIMD simulations without the need for enhanced sampling further supports their low activation barrier. These findings suggest that mineral surfaces, particularly those enriched in reactive NBOs, provide a kinetically privileged environment for CO_2_ activation, distinct from aqueous-phase pathways.

Structurally, NBOs are distributed throughout the olivine lattice, but those at the surface exhibit larger atomic volumes (209 Å^3^ vs. 121 Å^3^ in the bulk; Fig. 1e), reflecting reduced steric constraints and enhanced electronic polarizability. These properties increase the nucleophilicity of surface NBOs toward CO_2_. Local metal coordination further modulates this reactivity. Although Ca^2+^ and Mg^2+^ carry similar formal charges, Ca^2+^ has a much larger ionic radius and atomic volume (161–234 Å^3^ vs. 89 Å^3^ for Mg^2+^ on the surface; in Supplementary Table 1), leading to weaker metal–oxygen bonds. Consequently, Mg^2+^-coordinated NBOs are more electronically stabilized and less reactive than those coordinated to Ca^2+^. Lattice geometry also plays a key role. In CaMgSiO_4_, the vertical height difference (h) between NBOs and the surface is the largest, hindering H⁺ accessibility and suppressing hydrolysis. For CO_2_ uptake, the structural flexibility, quantified by the cation distance (d), is highest in Ca_2_SiO_4_ and lowest in Mg_2_SiO_4_ (Fig. 1f and Supplementary Fig. 1), enabling more effective accommodation of carbonate species in Ca-rich systems. Together, these findings show that CO_2_ reactivity in Ca-olivine arises from a synergistic interplay of pathway selectivity, electronic structure, and lattice-level geometric accessibility. Surface reactivity is governed by NBOs, whose nucleophilicity is strongly modulated by coordination with either Ca^2+^ or Mg^2+^.

### Surface carbonation facilitates cation dissolution

**Fig. 2:**
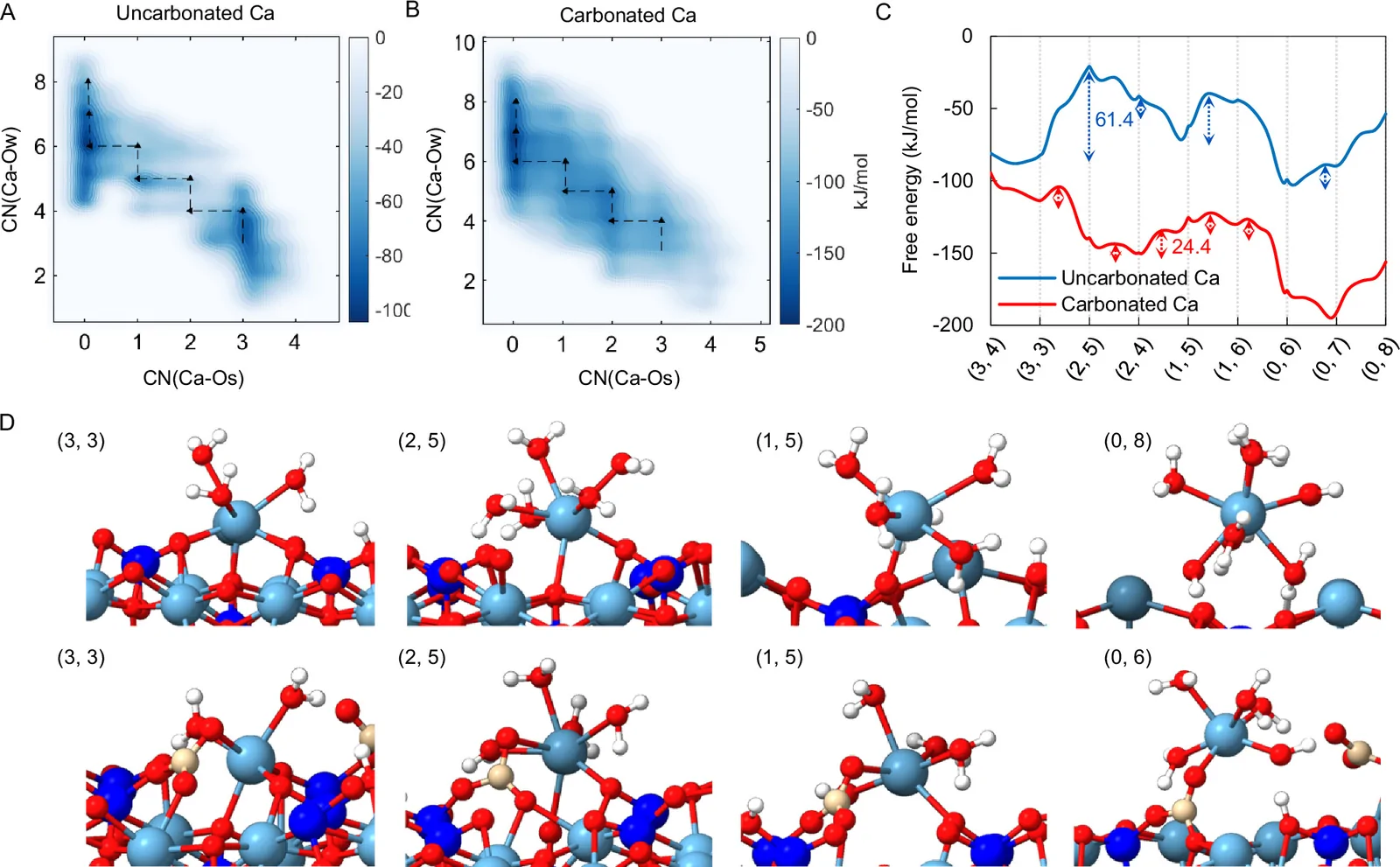
Dissolution behavior of uncarbonated and carbonated Ca in olivine. **A** Free energy surface of uncarbonated Ca dissolution. **B** Free energy surface of carbonated Ca dissolution. The black dashed line indicates the dissolution pathway. CN(Ca–Os) denotes the coordination number between Ca and silicate oxygen, while CN(Ca–Ow) denotes the coordination number between Ca and water oxygen. **C** Free energy evolutions of uncarbonated and carbonated Ca ions. **D** Snapshots of uncarbonated (top) and carbonated (bottom) Ca on Ca_2_SiO_4_ surface. The labels (m, n) above the snapshots denote the coordination state of Ca, where the first number, m, represents CN(Ca–Os) and the second number, n, represents CN(Ca–Ow). Calcium, silicon, oxygen, carbon, and hydrogen atoms are shown in light blue, dark blue, red, brown, and white, respectively. The energy barrier is reduced from 61.4 kJ/mol (uncarbonated) to 24.4 kJ/mol (carbonated), indicating that surface carbonation substantially accelerates Ca^2+^ dissolution. Based on transition state theory, this reduction leads to a dissolution rate increase of ~ 3.0 × 10^6^-fold.

Surface species control the mechanism and rate of silicate dissolution in dilute solutions^25^. To investigate the effect of surface carbonation on Ca^2+^ release, we performed MTD simulations to map the free energy surfaces for Ca^2+^ dissolution from Ca_2_SiO_4_ under both uncarbonated and carbonated conditions (Fig. 2a, b). In the uncarbonated system, Ca^2+^ follows a multistep pathway through intermediate coordination states, ultimately reaching the fully solvated state (0, 8). On carbonated surfaces, however, the pathway terminates earlier at (0, 6), due to Ca^2+^ coordination with CO_3_^2−^. The energy barrier at the rate-limiting step is reduced from 61.4 kJ/mol (uncarbonated) to 24.4 kJ/mol (carbonated), indicating that surface carbonation substantially accelerates Ca^2+^ release (Fig. 2c and Supplementary Fig. 2). Based on transition state theory, this reduction corresponds to a rate increase of ~ 3.0 × 10^6^-fold. In this analysis, the MTD-derived free-energy profiles are used primarily to compare relative detachment barriers under consistent simulation settings, rather than to determine absolute standard-state dissolution free energies^23^. Allowing for an uncertainty of ± 3 kJ/mol in the free-energy barrier difference between carbonated and uncarbonated Ca^2+^, the estimated dissolution rate enhancement ranges from 9.0 × 10^5^-fold to 1.0 × 10^7^-fold. This range is used in the following section to estimate the change in the apparent dissolution rate. Structural snapshots (Fig. 2d) show that CO_3_^2^^−^ coordination weakens Ca–O bonds within the mineral, facilitating ion detachment. Coordination with  CO_3_^2^^−^ delocalized electron density around Ca^2+^, weakening its coordination bonding to surrounding ligands and enabling easier dissolution into the aqueous phase. Mg^2+^ exhibits a higher dissolution free energy barrier than Ca^2+^^21^ due to the stronger Mg–O coordination bonds and the higher charge density associated with the smaller ionic radius of Mg^2+^. These factors hinder its release from the mineral lattice, consistent with the above observation of lower surface reactivity for Mg-olivine compared to Ca-olivine.

These results show that Ca_2_SiO_4_ not only possesses higher intrinsic surface reactivity but also benefits from a significant reduction in dissolution barrier driven by the changes in local coordination environments. This enhancement effect of surface carbonation is critical for accelerating cation release and sustaining carbonate precipitation. Unlike CaO and MgO, which suffer from passivating surface layers and thereby reduced reactivity for CDR^27^, silicate minerals such as Ca_2_SiO_4_ can maintain sustained reactivity under CO_2_ exposure. Notably, the enhanced cation dissolution may offset the passivation effect by exposing new NBOs sites. Thus, the renewal of surface reactivity by dissolution emerges as a key mechanism for enabling continuous carbonation, coupling surface reactions with dissolution–precipitation dynamics. It should be noted that the carbonation-induced reduction in the Ca dissolution barrier under neutral conditions and is applied in the next section for neutral or close to neutral conditions, while future simulations with controlled protonation states would be useful for resolving the additional influence of local interfacial pH.

### CO_2_ uptake kinetics under ambient conditions

**Fig. 3:**
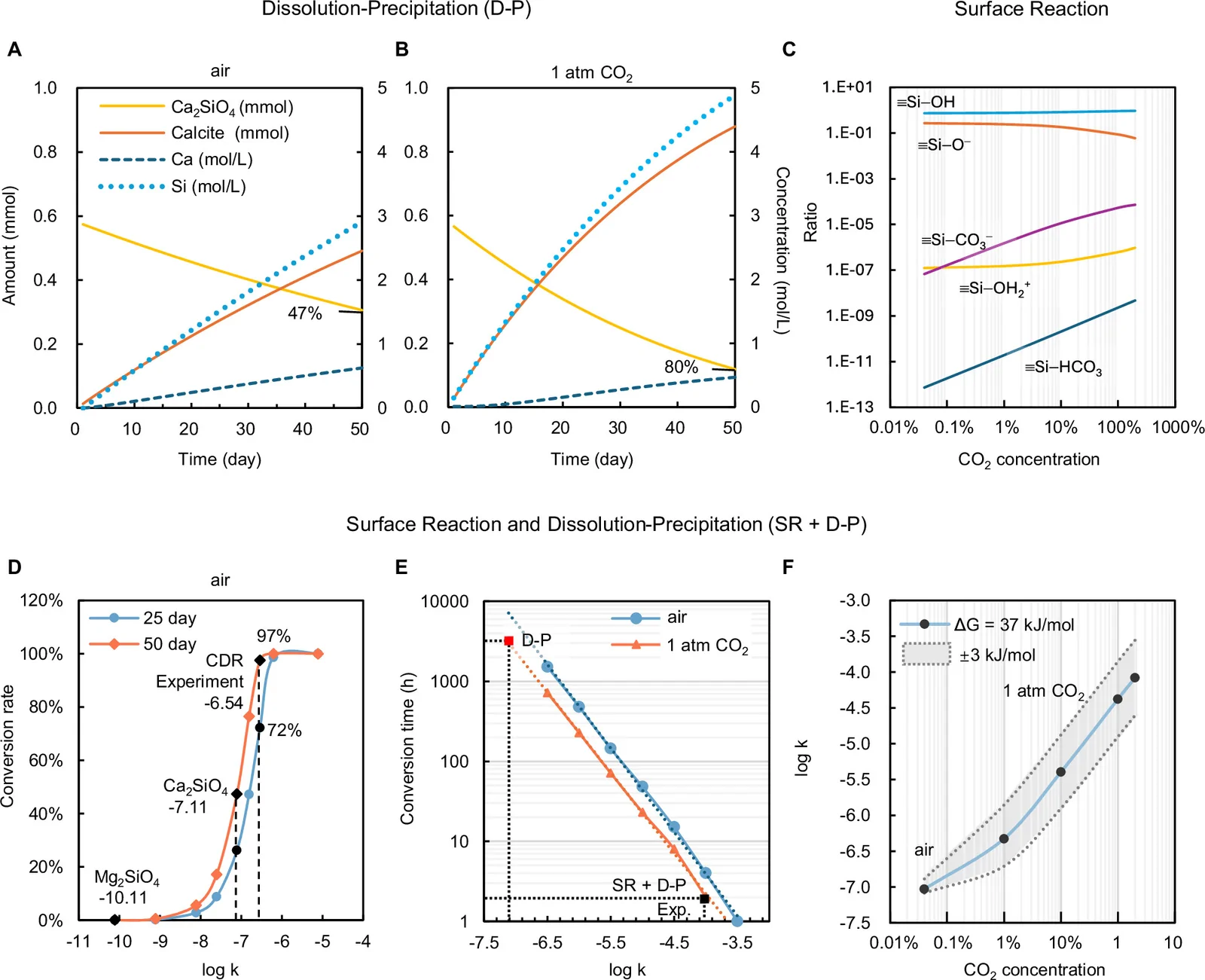
Carbonation kinetics of Ca-olivine governed by coupled dissolution-precipitation (D-P) and surface reactions (SR). **A** Time-dependent dissolution of Ca-olivine (Ca_2_SiO_4_) and calcite precipitation under air. **B** Time-dependent dissolution of Ca-olivine (Ca_2_SiO_4_) under 1 atm CO_2_. **C** Ratio of surface species of Ca_2_SiO_4_ with increasing CO_2_ concentration, showing the transition from ≡Si−O^−^ to ≡Si−CO_3_^−^ and ≡Si−HCO_3_. CO_2_ concentration is expressed relative to the partial pressure of CO_2_ in air (0.04 % at 1 atm). Thus, 100 % CO_2_ corresponds to 1 atm pCO_2_, and 200 % CO₂ represents 2 atm CO_2_. **D** Conversion rate of olivine as a function of dissolution rate constant k. **E** Conversion time of olivine as a function of dissolution rate constant k. The red and black squares represent the conversion times predicted by the D–P and combined SR + D–P mechanisms, respectively. The latter overlaps with the experimental conversion time of 2 h (Exp.). **F** The relationship between apparent dissolution rate constant k' and CO_2_ concentration. The gray area indicates the apparent dissolution rate change by ± 3 kJ/mol (from 34.0 kJ/mol to 40.0 kJ/mol). The figure shows that the dissolution-precipitation mechanism alone cannot reproduce the experiment. By considering the CO_2_ surface reaction, the apparent dissolution rate increases with log k’ increasing from − 7.11 to – 4.08, and the estimated conversion time is significantly reduced. This reproduces the experimental order of magnitude well, thereby helping to resolve the long-standing mismatch with experiments.

We now integrate the findings from AIMD and MTD simulation into geochemical modeling to understand the carbonation process of CDR materials. Figure 3 illustrates the carbonation behavior and kinetics of Ca_2_SiO_4_ under various CO_2_ concentration conditions consistent with experiments^1^. Dissolution–precipitation dynamics were evaluated under air and 1 atm CO_2_ conditions (Fig. 3a, b). As the reaction progressed, Ca and Si were released into the solution, accompanied by calcite (CaCO_3_) precipitation and gradual depletion of the Ca_2_SiO_4_ phase. Precipitation rates decreased over time, and the material conversion rates after 50 days were 47% and 80%, respectively, calculated based on the remaining amount of Ca_2_SiO_4_. Under 1 atm CO_2_, in addition to equilibrating the solution with the CO_2_ gas phase, the dissolution rate of olivine increases by about two times, as supported by experimental data (Supplementary Note 1). However, the conversion rate under 1 atm CO_2_ is significantly different from experimental results, where complete conversion occurred within 2 h. It is consistent with in situ carbon mineralization results where the fast field conversion rate is much faster than the rates predicted by dissolution-precipitation (D-P) kinetics without considering CO_2_-rich phase reactions with the rock matrix^16^.

Figure 3c presents the distribution of surface species, including CO_2_ surface reactions with Ca-olivine by surface complexation modeling. The ratio of surface species distribution after CO_2_ surface reaction ratios under varying CO_2_ concentrations (from 0.04%, 1%, 10%, to 100% at 1 atm, and 100% at 2 atm, balanced with N_2_), with equilibrium constants derived from AIMD calculations (Supplementary Note 2). Species including ≡Si−CO_3_^−^ and ≡Si−HCO_3_ are the product of CO_2_ surface reactions shown in the first section. The amount of ≡Si−CO_3_^−^ species is at the same level of ≡Si−OH_2_^+^ species, the ratio of which varies from 10^−7^ to 10^−5^ with increasing CO_2_ concentrations. Under atmospheric CO_2_ levels (0.04%), the small reaction ratio of Si with CO_2_ is consistent with the weak carbonation signatures in dry CDR materials after seven weeks of exposure in air^1^. Notably, this ultimately generates carbonated Ca^2+^, which dissolves faster than its uncarbonated counterpart, as shown by MTD simulations.

Figure 3d shows a strong correlation between the dissolution rate constant (log k) and conversion efficiency: in the range of –10 to – 6, a small increase in log k can lead to dramatic improvements in conversion rate. For instance, Mg-olivine with log k = – 10.11 showed negligible conversion, while Ca-olivine with log k = – 7.11 achieved 26.2% and 47.3% conversion after 25 and 50 days, respectively. These values remain lower than those observed in CDR experiments (72% and near 100%), which correspond to an apparent log k of –6.54. Based on the calculated ratio of carbonated Ca^2+^, apparent dissolution rates (log k’) were estimated to be – 7.03, – 6.33, – 5.39, – 4.38, and – 4.08 under increasing CO_2_ levels (0.04%, 1%, 10%, to 100% at 1 atm, and 100% at 2 atm) instead of the intrinsic log k of –7.11 for uncarbonated Ca_2_SiO_4_ (Fig. 3f and Supplementary Note 3). Figure 3e shows conversion time based on dissolution and precipitation kinetics under air and 1 atm 100% CO_2_, respectively. When Ca-olivine has an apparent log k’ of – 4.08 under 1 atm CO_2_, the predicted time for complete conversion is 2 h, which is much closer to the experimental result within 2 h instead of on the scale of several thousand hours based on the D-P scheme. This shows that predictions using the combined SR and D-P scheme are more accurate than those using the D-P scheme alone.

Collectively, these results demonstrate that while dry conditions enable initial surface carbonation, they are insufficient to sustain mineral transformation. Surface reaction facilitates a continuous sequence of surface reaction, cation release, and carbonate precipitation. This coupled mechanism is essential for efficient CDR and highlights the critical role of wet conditions in enabling full carbonation pathways under ambient conditions. The proposed surface reaction and dissolution-precipitation mechanism, when integrated with fluid flow in porous media, provides a framework for simulating subsurface mineralization and resolving the long-standing difficulty in accurately predicting in situ carbon mineralization processes. When reactive transport modeling was used to simulate CO_2_ injection into stacked Columbia River basalt reservoirs aiming to reproduce the Wallula project, it predicted that only 20% of injected CO_2_ would mineralize within 10 years^28^. However, when the dissolution/precipitation rate of pyroxene was increased by one order of magnitude, the model reproduced the field observation, showing 90% CO_2_ conversion within the same time frame. This suggests that the dissolution/precipitation rate of basaltic minerals are significantly higher than previously assumed. The enhanced rates can be attributed to surface reaction processes, which effectively increase the apparent dissolution rate constant.

## Discussion

Olivine offers a promising route for carbon dioxide removal (CDR) strategies. When applied to direct air capture (DAC), using Ca-olivine synthesized from silicates requires less than 1MWh tCO_2_^−1^, half the energy demand of leading DAC technologies^1^. Compared with the typical cost of enhanced weathering (US$80−180 t CO_2_^−1^)^6^ and ocean alkalinity enhancement (US$20−100 t CO_2_^−1^)^29^, carbon mineralization offers the lowest cost of US$20−40 t CO_2_^−1^, depending on basalt deposits^30^. Field demonstrations in Oman further show that even naturally occurring Mg-rich olivine enables some of the fastest carbon mineralization rates worldwide^10^.

This study uncovers the atomistic origins of the distinct reactivity between Ca^2+^ and Mg^2+^ in olivine-group silicates under ambient and subsurface conditions. By integrating ab initio molecular dynamics, Metadynamics and geochemistry modeling, we constructed a multiscale framework that links cation identity to surface carbonation, cation dissolution, and mineral transformation. The reactivity contrast between Ca- and Mg-olivine is governed by the behavior of nonbridging oxygens (NBOs), whose nucleophilicity is modulated by their coordinated metal ions. Specifically, differences in ionic radius, metal–oxygen bond strength, and local coordination flexibility between Ca^2+^ and Mg^2+^ alter the accessibility and reactivity of NBOs, the formation of metal coordinated hydroxyls, and the energetics of carbonate species. Our simulations reveal that Ca-terminated surfaces exhibit enhanced CO_2_ uptake via two distinct surface reaction pathways, NBOs activation and Ca–OH bicarbonate formation, both of which are less favored in Mg-bearing olivine due to tighter coordination and reduced lattice flexibility. Crucially, surface carbonation reduces the free energy barrier for Ca^2+^ dissolution by over 60%, thereby accelerating metal ion release by over six orders of magnitude. Following previous work on ion removal from solid–water interfaces, we interpret these pathway-based free-energy profiles as mechanistic descriptors of relative detachment barriers and associated kinetic changes^23^. In contrast, Mg^2+^ remains kinetically sluggish, underscoring its limited role in dynamic mineral transformation under ambient conditions.

Geochemical modeling further bridges simulation and experiment by reproducing the observed carbonation rates of Ca_2_SiO_4_ under varied CO_2_ concentrations. Although surface carbonation is limited under completely dry conditions, this constraint is offset by accelerated Ca^2+^ dissolution during subsequent wet phases, enabling a self-sustaining surface reaction and dissolution–precipitation cycle. The apparent dissolution rate constant (log k’) serves as a key integrative descriptor that links atomistic-scale energetics to macroscale carbonation efficiency. Unlike CaO or MgO, which suffer from surface passivation upon carbonation, silicate-based materials such as Ca_2_SiO_4_ maintain sustained reactivity due to the dynamic interplay between surface reactions and dissolution-precipitation. These insights provide a mechanistic foundation for designing silicate materials with enhanced reactivity toward CO_2_ under ambient conditions.

The role of CO_2_ surface reactions may be underestimated if they are considered only under very water-limited conditions. Under extremely low water content, CO_2_ surface reactions may occur on mineral surfaces, but the subsequent dissolution and precipitation processes are limited. As a result, their contribution to measurable carbonation conversion can be difficult to detect experimentally and considered as minimal. In contrast, we propose that under wet or partially wet conditions, CO_2_ surface reactions can proceed continuously together with the dissolution–precipitation process. By reducing the dissolution barrier and enhancing the apparent dissolution rate as a function of CO_2_ concentration, surface carbonation can effectively accelerate cation release, indicating that CO_2_ surface reactions serve as an important additional mechanism for rapid carbon mineralization. The local water condition in the referenced experiments was not strictly controlled throughout the reaction, because water was added when the material appeared dry. Therefore, the system may have transitioned between aqueous and water-film conditions ( < 0.7 g/g)^31^ during carbonation. This introduces uncertainty into the prediction of carbonation conversion time, because water-film thickness can influence metal ion dissolution and the carbonation-induced reduction in dissolution free energy. Such sensitivity is consistent with previous demonstrations that pathway-based ion-release free energies at solid–water interfaces depend on the definition of the solution state and the configurational volume sampled by the detached ion^23^. Future studies combining controlled water/material-ratio experiments and simulations will be needed to quantitatively connect water-film thickness, dissolution energetics, and olivine carbonation kinetics.

The reaction mechanism identified for Ca-olivine CDR materials is remarkably consistent with that observed in basaltic carbon mineralization systems. Our result shows that both surface Ca^2+^ and inner Ca^2+^ facilitate the reaction of CO_2_ with NBOs in olivine. It implies that Mg-rich basalt reservoirs could also benefit from trace amounts of Ca^2+^ through a disproportionate amplification of carbonation kinetics. Although Ca^2+^ constitutes only 0–1 wt% of igneous olivine^12^, its reactivity exceeds that of Mg^2+^ by up to three orders of magnitude (log k’). Moreover, Ca is commonly enriched at the rims^32^, indicating that it may play a key role in initial dissolution and early-stage mineralization. Olivine can also benefit from additional Ca^2+^ released by coexisting minerals such as pyroxene and plagioclase. The adsorption of this dissolved Ca^2+^ onto olivine surfaces may further enhance its reactivity relative to pure Mg-olivine. Fe is another major cation in natural olivine. In this study, however, we focus first on Ca and Mg to isolate the effect of interfacial carbonation on dissolution kinetics. This simplification is also consistent with the experimental system used for validation, in which Fe was not explicitly considered. Quantifying the influence of Fe on interfacial carbonation pathways and dissolution kinetics, therefore, represents an important direction for future work. It should be noted that natural basaltic systems are considerably more complex than the simplified systems considered here. They commonly contain mixed mineral assemblages, Fe-bearing olivine, Al-bearing phases, impurities, heterogeneous surfaces, variable reactive surface areas, fluid-flow effects, and spatial variations in pH. The effect of pH was considered only in the geochemical modeling, whereas explicitly incorporating pH effects into molecular dynamics and metadynamics simulations^33^ remains an important direction for future investigation. This study highlights the critical role of surface reactions in driving carbon mineralization, bridging the gap between slow carbonate precipitation kinetics and fast carbon mineralization field results^16^. The multiscale framework developed here offers a generalizable approach across multicomponent silicates, basaltic rocks, and industrial byproducts, offering guidance for scalable CDR strategies under diverse environmental settings. We hope that this study can be stimulating for further research in field applications.

## Methods

### DFT parameters

All ab initio molecular dynamics (AIMD) simulations used the CP2K/QUICKSTEP program (version 2023.2)^34^. The exchange and correlation terms were described by the Perdew-Burke-Ernzerhof (PBE) functional^35^. Dispersion corrections were accounted for using Grimme’s third generation DFT-D3 correction^36^, because long-range van der Waals interactions are important for describing CO_2_ and H_2_O adsorption at mineral interfaces, and PBE-D3 has been widely applied in simulations of CO_2_–H_2_O–mineral systems^17,22,37–40^. Norm-conserving pseudopotentials were used to address the core electrons^41^, and a double-zeta quality basis set was used to address the valence part^34^. AIMD simulations were performed in the canonical (NVT) ensemble using periodic boundary conditions with a timestep of 1 fs^17^. For hydrolysis and carbonation reactions, the temperature was controlled at 323 K for 10 ps using the Nosé–Hoover thermostat^42^. A 280 Ry plane-wave cutoff value was used, and the spin polarization was not considered in the calculations as in previous research^37,39^. Bader charge analysis was performed to examine the electronic structure of metal–oxygen coordination environments^43^, while nudged elastic band (NEB) calculations were used to evaluate the reaction energetics of Ca^2+^ and Mg^2+^ with CO_2_ and H_2_O for comparison of their reactivity^44^.

### Simulation models

We constructed slab models of Ca_2_SiO_4_, CaMgSiO_4_, and Mg_2_SiO_4_ (forsterite) for surface reaction simulations. For Ca_2_SiO_4_, the orthorhombic unit cell (a = 5.0762 Å, b = 11.2136 Å, c = 6.7583 Å, α = β = γ = 90°) was expanded to a 2 × 1 × 2 supercell and cleaved along the (010) direction^45^. For CaMgSiO_4_, we cleaved the surface along the (100) direction to expose the Ca termination. The unit cell parameters are a = 11.1098 Å, b = 6.3894 Å, c = 4.8281 Å with α = β = γ = 90°, and a 1 × 2 × 3 supercell was employed^46^. For Mg_2_SiO_4_, which also crystallizes in the orthorhombic system (a = 4.763 Å, b = 10.24 Å, c = 5.999 Å)^47^, we adopted a 3 × 1 × 2 supercell cleaved along the (010) surface, exposing the M2 termination, which has been shown to be the most stable configuration^18,48^. In all three structures, the M2 sites were exposed on the surface (Supplementary Fig. 3), providing a consistent basis for comparing reactivity across different compositions. A 7 Å-thick water layer was initially used for hydrolysis simulations and then substituted by CO_2_ at a density corresponding to 323 K and 10 MPa, which is the typical condition of subsurface carbon mineralization. This 0.7 nm interfacial layer was chosen to provide sufficient H_2_O or CO_2_ molecules for hydrolysis and carbonation reactions within the simulation cell. In the carbonation simulations, different surface water densities were considered when assessing the relative reactivity of the olivine compositions. This allows direct comparison of surface reactions between water and CO_2_ within a tractable AIMD simulation window.

### Metadynamics

To investigate the dissolution behavior of uncarbonated and carbonated Ca, two surface models of Ca_2_SiO_4_ were used for well-tempered metadynamics simulations^49^: the hydrolyzed surface obtained after hydrolysis simulation, and the carbonated surface after carbonation simulation. A 14 Å-thick water layer was added above the surface and pre-equilibrated in the NVT ensemble using the Nosé–Hoover thermostat at 323 K. The positions of silicon atoms in the middle two layers of the slab were fixed to prevent structural drift during the simulation. Coordination number (CN) with mineral oxygens, and CN with water or CO_3_^2−^ oxygens were used as collective variables, respectively. The expression of the switch function used for calculating the CN is as follows:

2 $${{{\rm{CN}}}}\left(X-Y\right)=\frac{1}{{N}_{X}}{\sum }_{{i}_{X}}{\sum }_{{j}_{Y}}\frac{1-{\left(\frac{{r}^{{ij}}}{{R}_{0}}\right)}^{{{{\rm{nn}}}}}}{1-{\left(\frac{{r}^{{ij}}}{{R}_{0}}\right)}^{{{{\rm{nd}}}}}}$$

Where nn = 16, nd = 32, and *R*_0_ = 3.2 Å for Ca-O^22^. The Gaussian hills were deposited every 30 timesteps with an initial height of 2.5 kJ/mol and a width of 0.2 for both CVs. The system temperature was 323 K, and the bias factor was 20 for Ca ions. The simulation temperature of 323 K was chosen to ensure consistency with AIMD runs. These metadynamics parameters were selected following previous applications of well-tempered metadynamics to ion dissolution from mineral surfaces and solid–water interfaces^22,23,40^. The dissolution energy difference between 323 K and ambient temperature (298.15 K) is considered negligible in molecular dynamics simulations. The time evolution of CVs and convergence tests are shown in Supplementary Figs. 4−7.

### Geochemical modeling

#### Surface complexation modeling

A surface complexation model (SCM) was developed to describe the reaction with water and CO_2_ at the Ca_2_SiO_4_ surface using PHREEQC^50^. The same termination of Ca-olivine as the AIMD simulation model was used, with surface concentrations of the two initial species ≡Si−O^−^ → Ca^+^<(nonbridging oxygen, abbreviated as ≡Si−O^−^) and > CaOH shown in Supplementary Table 2. The six reactions included are shown in Supplementary Table 3. Reaction (1) describes the protonation of nonbridging oxygens (NBOs) on the Ca-olivine surface. Reaction (2) describes the secondary protonation of NBOs on the Ca-olivine surface. The equilibrium constant log K of reaction (1) and (2) is referenced from wollastonite (CaSiO_3_)^51^, where the NBO environments are structurally similar to those in Ca-olivine. Reaction (3) and (4) describe the hydrolysis reaction of surface Ca^2+^, the reaction stability constants were adopted from those of CaO^51^, an assumption proved valid by a previous study^52^. The surface reaction with CO_2_ was described by reaction (5). Only pathway 1 is included here because it is the major and generalized reaction across all mineral surfaces^17^. The equilibrium constants of reaction (5) were determined by AIMD results, and the calculation method is shown in Supplementary Note 2. Reaction (6) involves the protonation and deprotonation between surface CO_3_ and HCO_3_ species, the equilibrium constant value of which was taken from the similar reactions in calcium carbonate minerals^53,54^. The initial amount of Ca_2_SiO_4_ and water were 0.1 g and 0.1 mL, respectively, consistent with experiments^1^. The simulation was designed to represent the exposure of Ca-olivine to air. Water was equilibrated with CO_2_ at concentrations ranging from 0.04% (air level) to 100% CO_2_ at 1 atm, and 100% CO_2_ at 2 atm, with nitrogen serving as the balance gas.

#### Dissolution and precipitation kinetics

The dissolution and precipitation simulations of Ca-olivine were carried out using PHREEQC^50^ and the LLNL database. The dissolution rate constant (log k) of forsterite is -10.11 at 25 °C and pH 7.19, and the dissolution kinetics of Ca-olivine follows the approach by previous studies^55^. Under the same condition, the dissolution rate of Ca-olivine is three orders of magnitude higher than that of forsterite^56^, therefore log k = − 7.11 is used for Ca-olivine. The effect of pH on dissolution rate is considered with details provided in Supplementary Note 1. Kinetic simulations were conducted at 25 °C, 1 atm or 2 atm, and different concentrations of CO_2_. Mineral precipitation reactions were assumed to be controlled by their saturation state in solution and instant equilibration. The water ratio is 1 g material/g of water, the same as experimental setting^1^. The surface area of CDR materials is reported to be less than 1 m^2^/g without a specific value, and 0.01 m^2^/g is used instead. Evaporation was considered by linearly reducing the amount of water for 24 h, and simulation results show that the precipitation amount is only minimally affected by evaporation (Supplementary Fig. 8). Therefore, it is not considered in the simulation in the main text.

## Supplementary information

Supplementary Information for Publication

Supplementary Data

Transparent Peer Review file

## Data Availability

All data presented in this study are provided in the Supplementary Information/Supplementary Data files.
