# Supplementary material for: Surface carbonation governs olivine reactivity for rapid CO2 mineralization
Source: Nat Commun. 2026 Oct 14;17:11616. doi: 10.1038/s41467-026-78548-z (PMC13755434; doi:10.1038/s41467-026-78548-z)
Supplement: Supplementary file 1 [file 41467_2026_78548_MOESM1_ESM.pdf]

Supporting Information

Surface carbonation governs olivine reactivity for rapid CO_2 mineralization

Zihua Shao [1], Yunfeng Liang [1,2*], Gyuhwan Jo [2], Keisuke Nakamura [2], and Takeshi Tsuji [1*]

[1] Department of Systems Innovation, School of Engineering, The University of Tokyo, Tokyo, 113-8656, Japan

[2] e-Technology Innovation Center, ENEOS Xplora Inc., Tokyo, 106-0041, Japan

*Corresponding author

E-mail: liang@sys.t.u-tokyo.ac.jp (Y.L.)

E-mail: tsuji@sys.t.u-tokyo.ac.jp (T.T.)

This file includes:

Supplementary Notes 1 to 3

Supplementary Figures 1 to 9

Supplementary Tables 1 to 3

Supplementary References 1 to 7

Supplementary Note 1. Effect of pH on olivine dissolution rate

When the aqueous phase is equilibrated with the partial pressure of CO_2 in air, the pH of water is approximately 7, and the dissolution rate constant under neutral conditions is therefore adopted. When the aqueous phase is equilibrated with pure CO_2 at 1 atm, the pH during dissolution ranges from 5.7 to 5.1 (Supplementary Figure 9).

The dissolution rate of olivine can be expressed as follows [1]:

$$R(\mathrm{mol}\cdot\mathrm{cm}^{-2}\cdot\mathrm{s}^{-1}) = 9.07\times10^{-12}a_{\mathrm{H}^{+}}^{0.54} + 5.25\times10^{-15} + 2.33\times10^{-17}a_{H^{+}}^{-0.31}$$

Although this relationship is based on forsterite, it is used here to estimate the enhanced dissolution rate of Ca olivine under acidic environment, as olivine minerals exhibit similar dissolution behavior [2]. When pH = 7, log R = –13.99; when pH = 5.1, log R = –13.65, corresponding to an increase of approximately 0.33 orders of magnitude in the dissolution rate. That is, the dissolution rate increases by about two times (2.14 times).

Supplementary Note 2. Calculation method of reaction stability constants

For CO_2 surface reaction pathway 1 (reaction (5) in supplementary table 3), the reaction stability constant can be calculated as follows:

$$K_5 = \frac{\{\equiv \mathrm{Si}-\mathrm{CO}_3^- \rightarrow \mathrm{Ca}^+ <\}}{\{\equiv \mathrm{Si}-\mathrm{O}^- \rightarrow \mathrm{Ca}^+ <\} \cdot a_{\mathrm{CO}_2}}$$

where, $\{\equiv \mathrm{Si}-\mathrm{CO}_3^- \rightarrow \mathrm{Ca}^+ <\}$ is the surface concentration of reacted nonbridging oxygen, and $\{\equiv \mathrm{Si}-\mathrm{O}^- \rightarrow \mathrm{Ca}^+ <\}$ is the surface concentration of nonbridging oxygen. The surface concentration of species is calculated based on the configuration of ab initio molecular dynamics simulations. The density of surface species is based on ab initio simulation and listed in Table S4.

The activity of CO_2 is gas state in simulation, which can be calculated as follows:

$$a_{\mathrm{CO}_2} = \frac{f_{\mathrm{CO}_2}}{f^{\circ}}$$

where f_{CO_2} is the fugacity of CO_2, and f° is the standard-state fugacity (1 bar). For the fugacity of CO_2:

$$f_{\mathrm{CO}_2} = \phi P$$

where ϕ is fugacity coefficient which can be calculated by Peng-Robinson equation [3]. For pure CO_2 gas in simulation at 323 K and 10 MPa, $\phi \approx 0.618$, and f_{CO_2}=61.8 bar.

$$K_5^{(\mathrm{g})} = \frac{\{\equiv \mathrm{Si}-\mathrm{CO}_3^- \rightarrow \mathrm{Ca}^+ <\}}{\{\equiv \mathrm{Si}-\mathrm{O}^- \rightarrow \mathrm{Ca}^+ <\} \cdot a_{\mathrm{CO}_2}} = \frac{1.82}{4.74 \times 61.8}$$

where the density of initial and reacted nonbridging oxygen on Ca-olivine surface is based on the model after hydrolysis simulation, which is slightly different from Table S1 (density of pristine surface).

$$\log K_5^{(\mathrm{g})} = -2.21$$

The activity of aqueous CO_2 is:

$$a_{\mathrm{CO_{2(aq)}}} = \frac{\gamma_{\mathrm{CO_2}} m_{\mathrm{CO_2}}}{m^{\circ}}$$

where m_{CO_2} is the molarity of CO_2 in water; γ_{CO_2} is the activity coefficient, for ideal solution γ_{CO_2}= 1; m° is standard state molarity, 1 mol/kg.

$$a_{\mathrm{CO_{2(aq)}}} = \gamma_{\mathrm{CO_2}} K_{\mathrm{H}} f_{\mathrm{CO_2}}$$

where $\gamma_{\mathrm{CO_2}}$ is activity coefficient of CO_2, which can be approximated as 1 for dilute solutions; K_{H} denotes Henry's law constant ($\mathrm{mol \cdot L^{-1} \cdot atm^{-1}}$). At 25°C, K_{H} = 0.34 mol/(L·MPa) [4].

$$K_5^{(\mathrm{aq})} = K_5^{(\mathrm{g})} \frac{a_{\mathrm{CO_2(g)}}}{a_{\mathrm{CO_2(aq)}}} = K_5^{(\mathrm{g})} \frac{1}{K_{\mathrm{H}}}$$

$$\log K_5^{(\mathrm{aq})} = \log K_5^{(\mathrm{g})} - \log K_{\mathrm{H}} = -2.21 - \log K_{\mathrm{H}} = -1.74$$

Supplementary Note 3. Calculation of apparent dissolution rate constant (log k')

Apparent dissolution rate constant (log k') is the dissolution rate constant of Ca_2SiO_4 considering the promotion effect of carbonated Ca. The ratio of carbonated Ca is calculated from the SCM results. For carbonated surface:

$$R'_{\mathrm{Ca}} = k_{\mathrm{uncarbonated}}\{>\mathrm{CaOH}_2^+\} + k_{\mathrm{carbonated}}\{>\mathrm{CaCO}_3^-\}$$

where, $\{>\mathrm{CaOH}_2^+\}$ is the surface concentration of uncarbonated Ca, and $\{>CaCO_3^-\}$ is the surface concentration of carbonated Ca.

$$\frac{R'_{\mathrm{Ca}}}{R_{\mathrm{Ca}}} = \frac{k_{\mathrm{uncarbonated}}\{>\mathrm{CaOH}_2^+\} + k_{\mathrm{carbonated}}\{>\mathrm{CaCO}_3^-\}}{k_{\mathrm{uncarbonated}}(\{>\mathrm{CaOH}_2^+\} + \{>\mathrm{CaCO}_3^-\})}$$

$$\frac{R'_{\mathrm{Ca}}}{R_{\mathrm{Ca}}} = \frac{\{>\mathrm{CaOH}_2^+\} + \frac{k_{\mathrm{carbonated}}}{k_{\mathrm{uncarbonated}}}\{>\mathrm{CaCO}_3^-\}}{\{>\mathrm{CaOH}_2^+\} + \{>\mathrm{CaCO}_3^-\}}$$

where, R_{Ca} is the dissolution rate of uncarbonated surface, R'_{Ca} is the dissolution rate of carbonated surface.

Therefore, the log k' can be calculated by the following method:

$$\log k' = \log k \frac{\{>\mathrm{CaOH}_2^+\} + \frac{k_{\mathrm{carbonated}}}{k_{\mathrm{uncarbonated}}}\{>\mathrm{CaCO}_3^-\}}{\{>\mathrm{CaOH}_2^+\} + \{>\mathrm{CaCO}_3^-\}}$$

where, $\frac{k_{carbonated}}{k_{uncarbonated}}$ is calculated by the free energy barrier difference in metadynamics simulation, $\{>\mathrm{CaOH}_2^+\}$ and $\{>\mathrm{CaCO}_3^-\}$ are the surface concentration of uncarbonated and carbonated Ca, respectively, as calculated by SCM. The value of log k is -7.11, which is the dissolution rate of pristine Ca_2SiO_4.

Supplementary Figure 1.

Configurations of CO_2 molecules reacted with nonbridging oxygens.

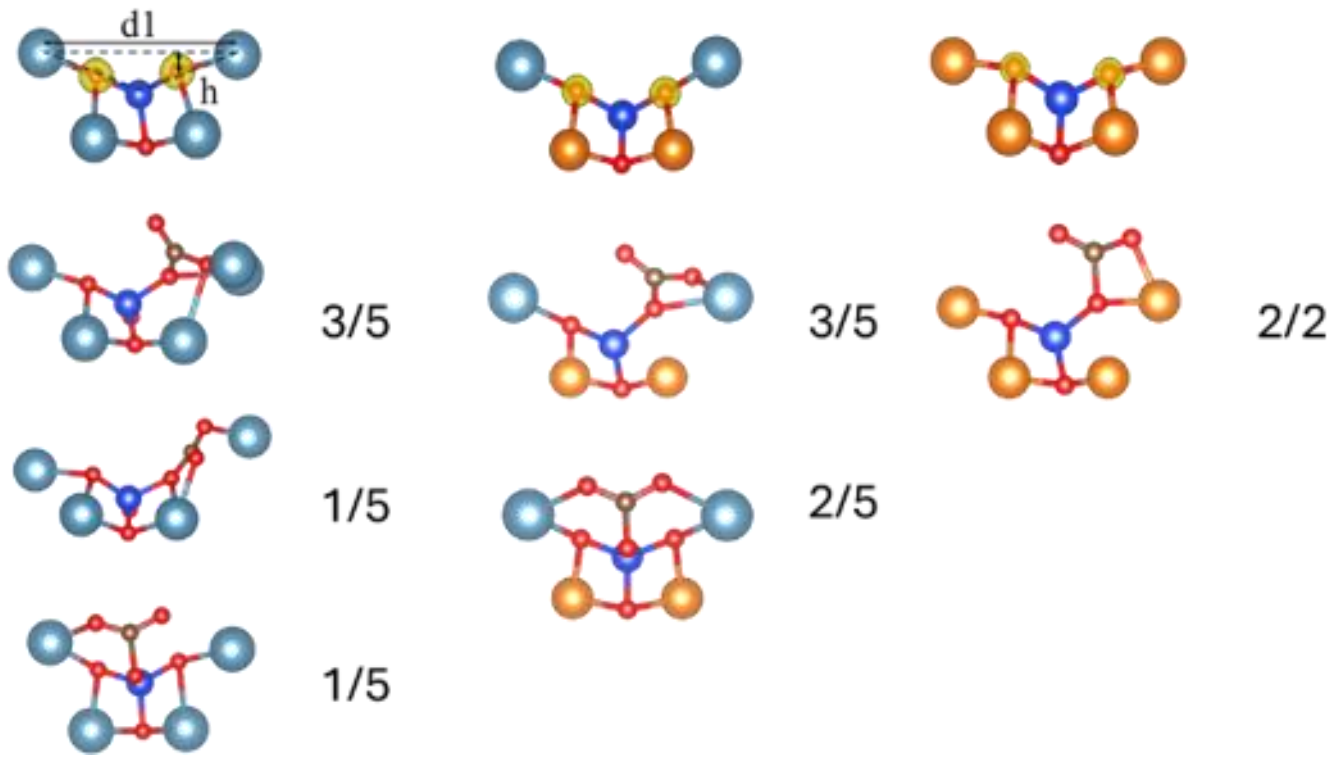

Supplementary Figure 2.

Free energy levels of states and transition states (TSs) along the dissolution pathway of uncarbonated and carbonated Ca^{2+}. The free energies of the initial states for both pathways were set to zero for clarity.

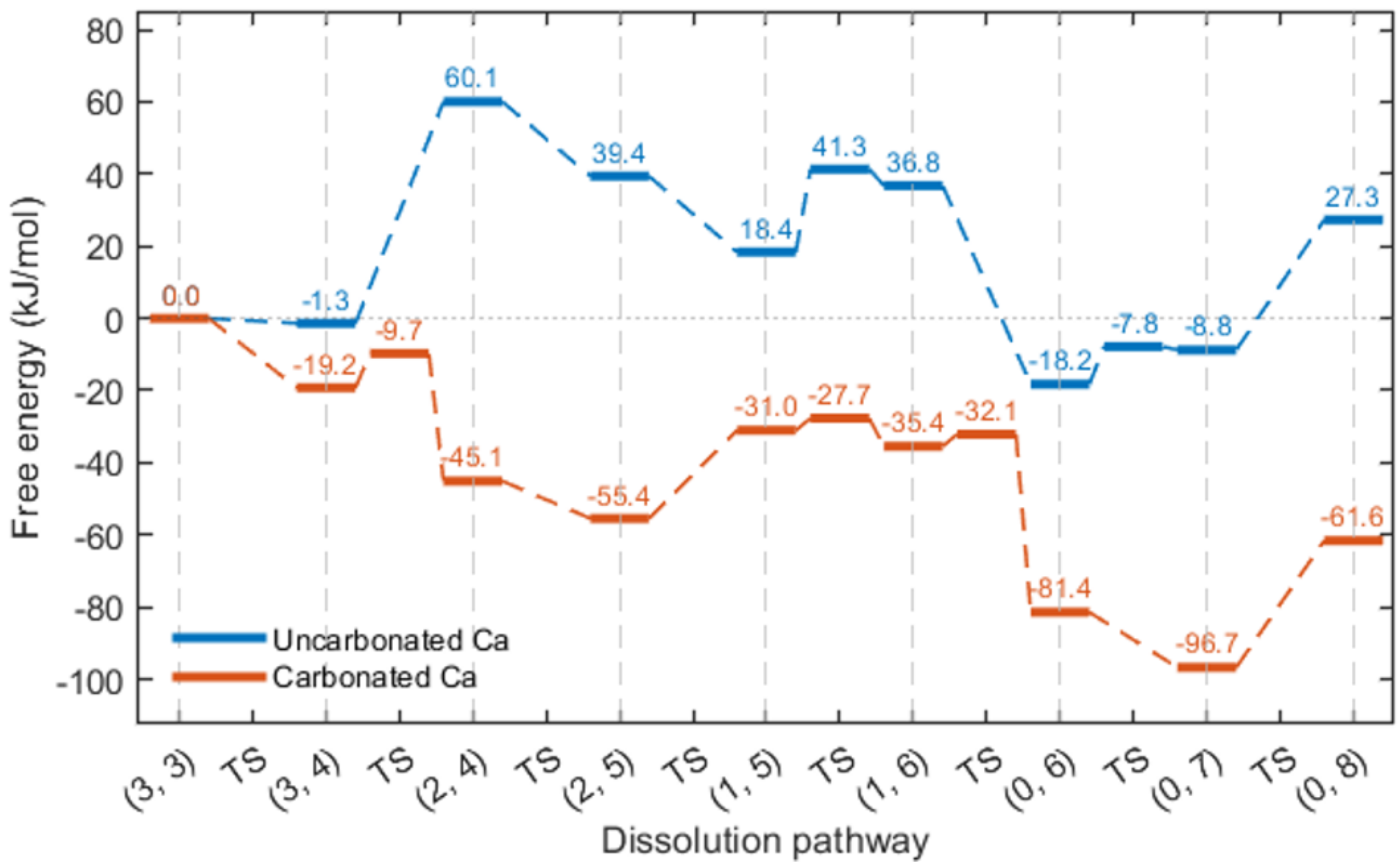

Supplementary Figure 3.

M1 and M2 cation sites in Ca_2SiO_4, $CaMgSiO_4$, and Mg_2SiO_4 mineral structures. The oxygen atom on the left is bonded to a silicon atom on the right side of the unit cell through periodic boundary conditions and thus is not an isolated species.

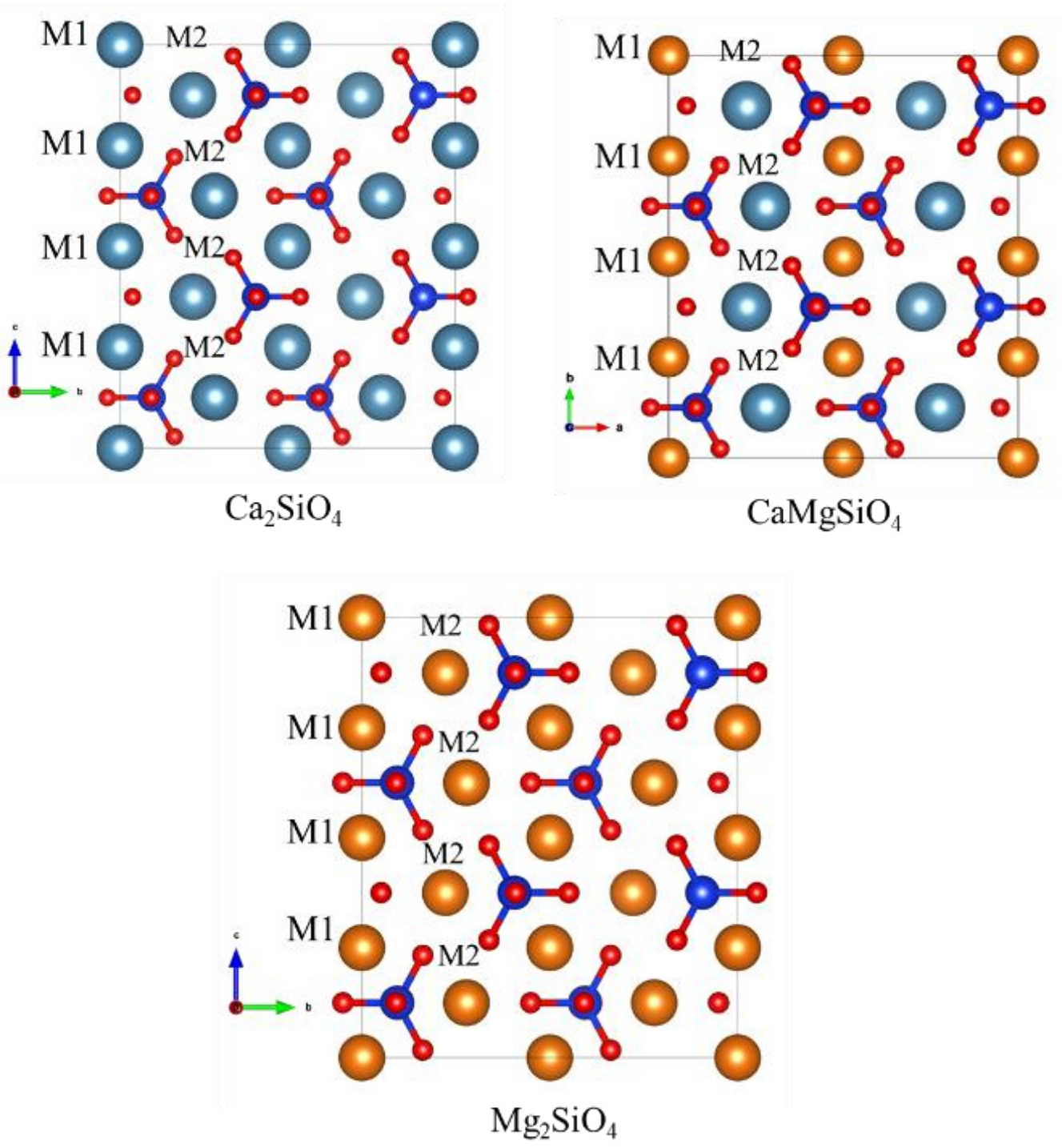

Supplementary Figure 4.

Time evolution of CN(Ca-Os), CN(Ca-Ow) and the Gaussian hill height during the well-tempered metadynamics simulation for uncarbonated Ca dissolution.

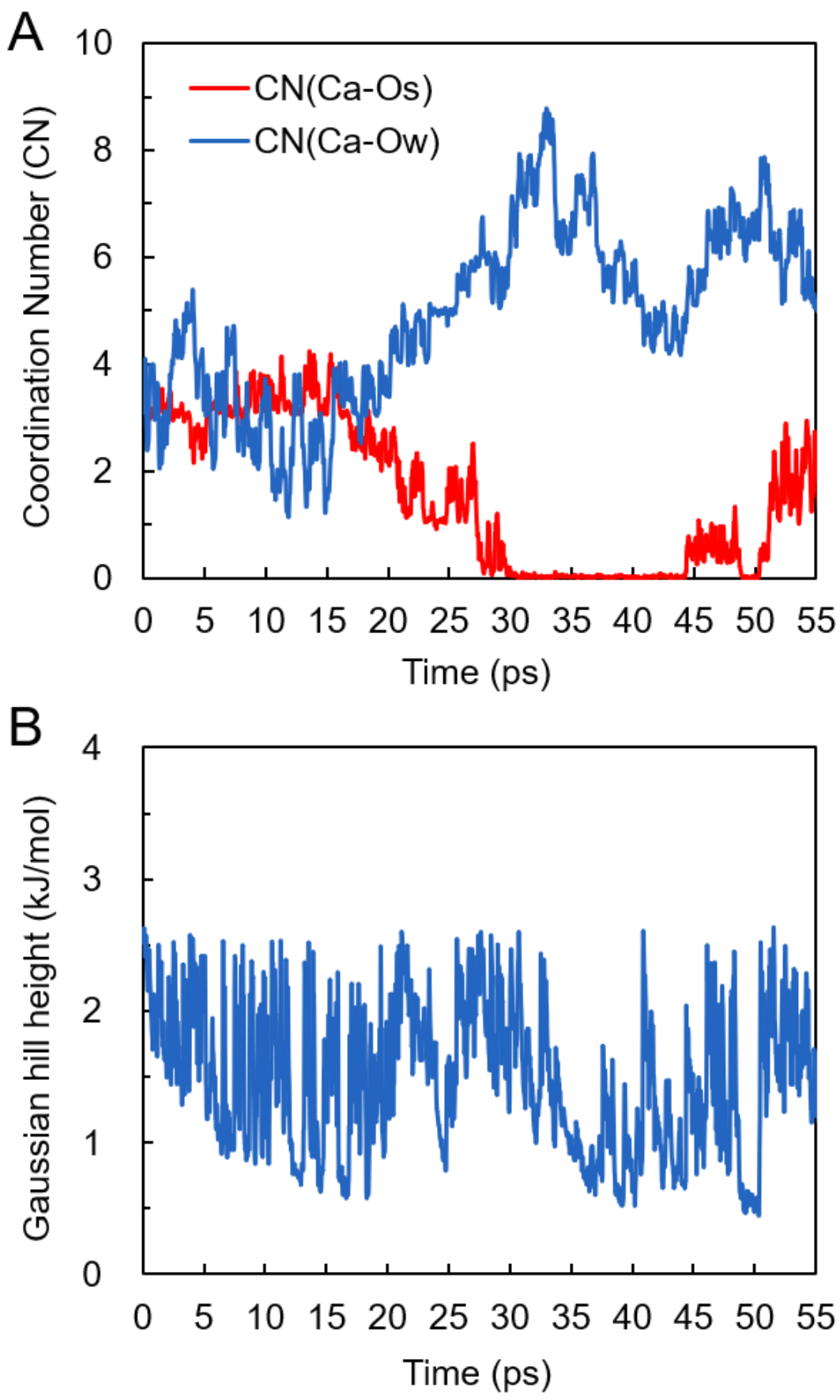

Supplementary Figure 5.

Convergence tests for well-tempered metadynamics simulation of uncarbonated Ca dissolution. (A, B) The free energy surface as a function of the CN(Ca-Os) or CN(Ca-Ow) every 3 ps (100 Gaussian kernels deposited) along the simulation time. (C) The free energy surface along the dissolution pathway every 3 ps. (D) Free energy barrier variations along the simulation time.

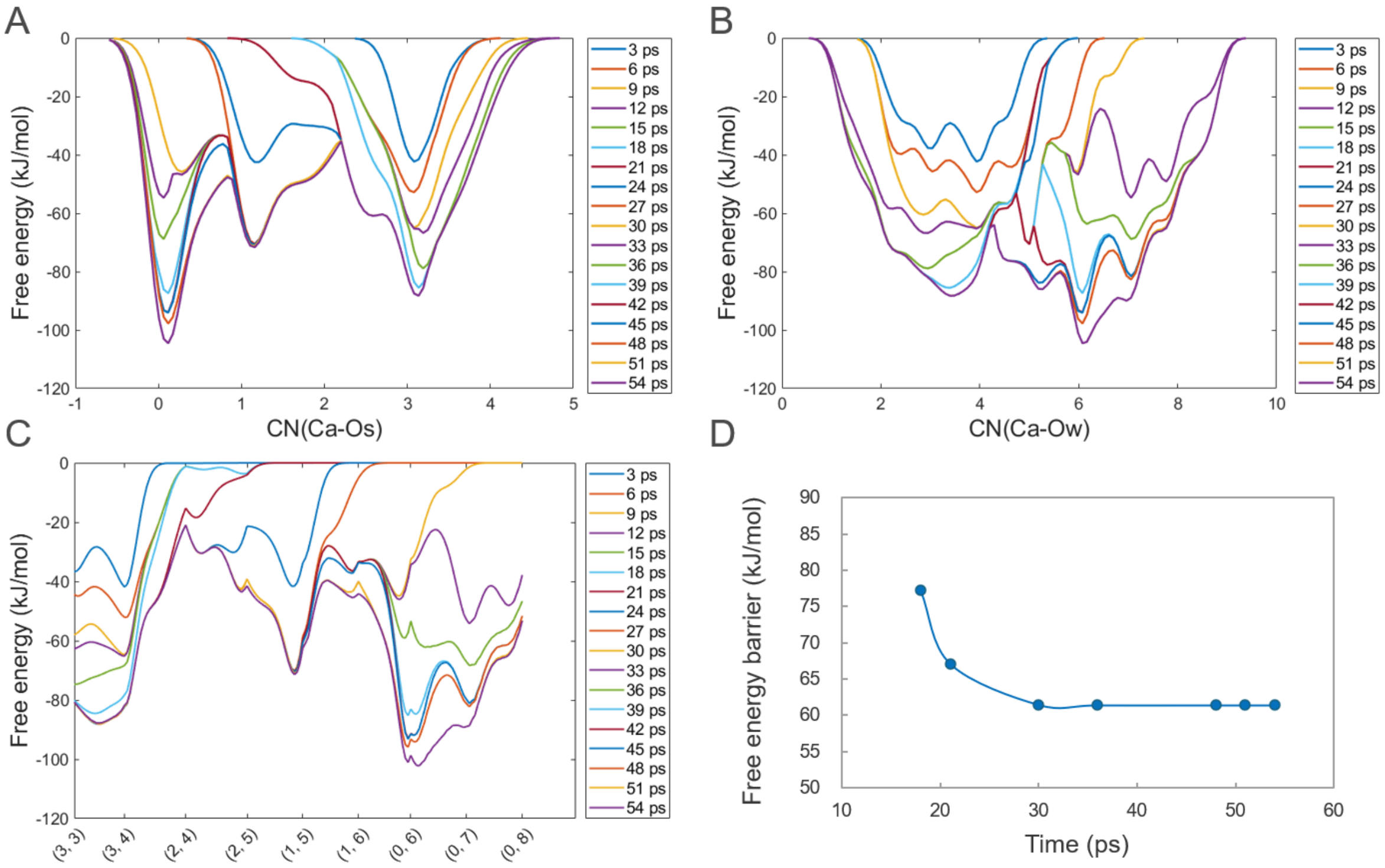

Supplementary Figure 6.

Time evolution of CN(Ca-Os), CN(Ca-Ow) (A) and Gaussian hill height (B) during the well-tempered metadynamics simulation for carbonated Ca dissolution.

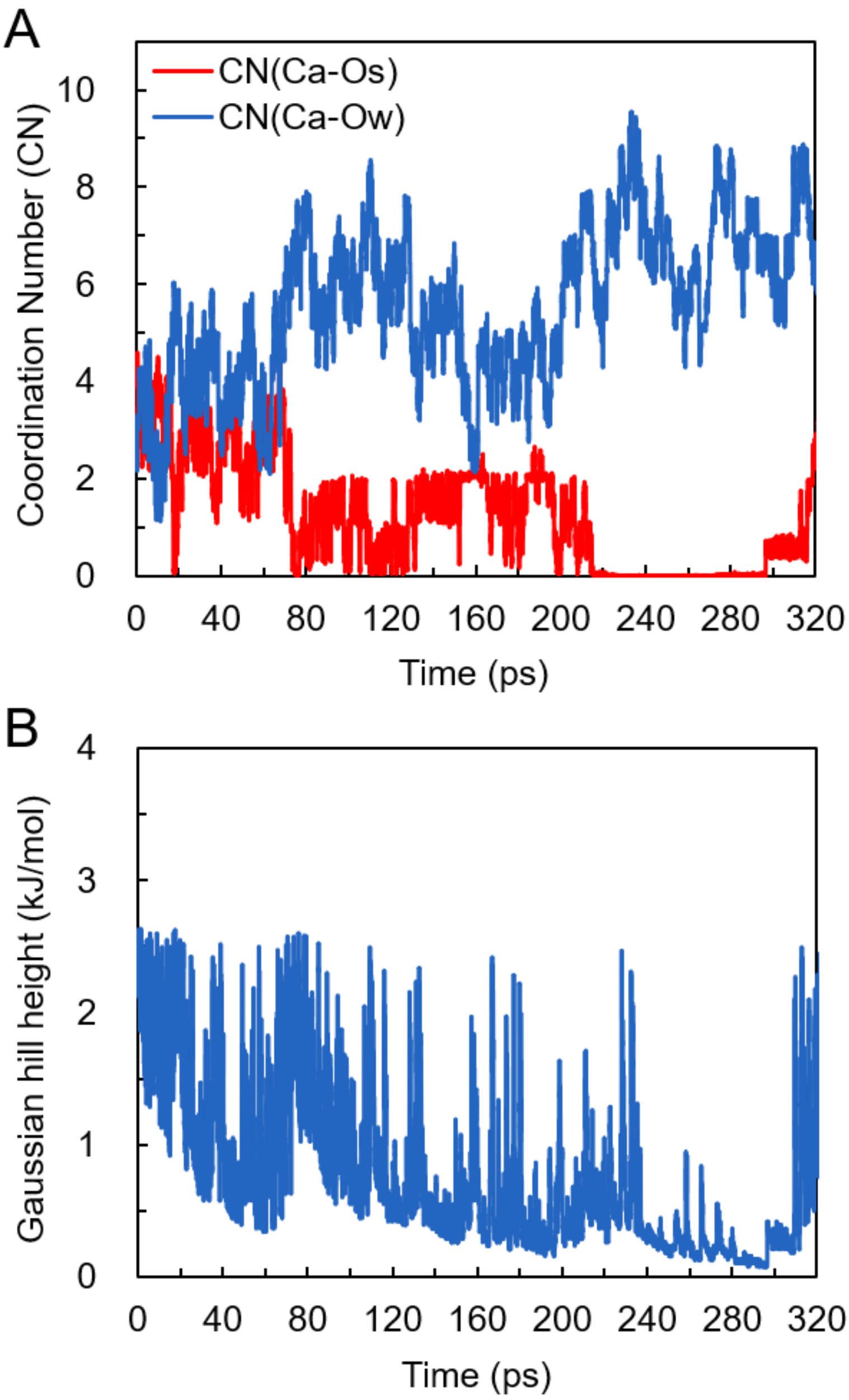

Supplementary Figure 7.

Convergence tests for well-tempered metadynamics simulation of carbonated Ca dissolution. (A, B) The free energy surface as a function of the CN(Ca-Os) or CN(Ca-Ow) every 30 ps during the first 300 ps and every 3 ps (100 Gaussian kernels deposited) during the last 18 ps. (C) The free energy surface along the dissolution pathway along the simulation time. (D) Free energy barrier variations along the simulation time.

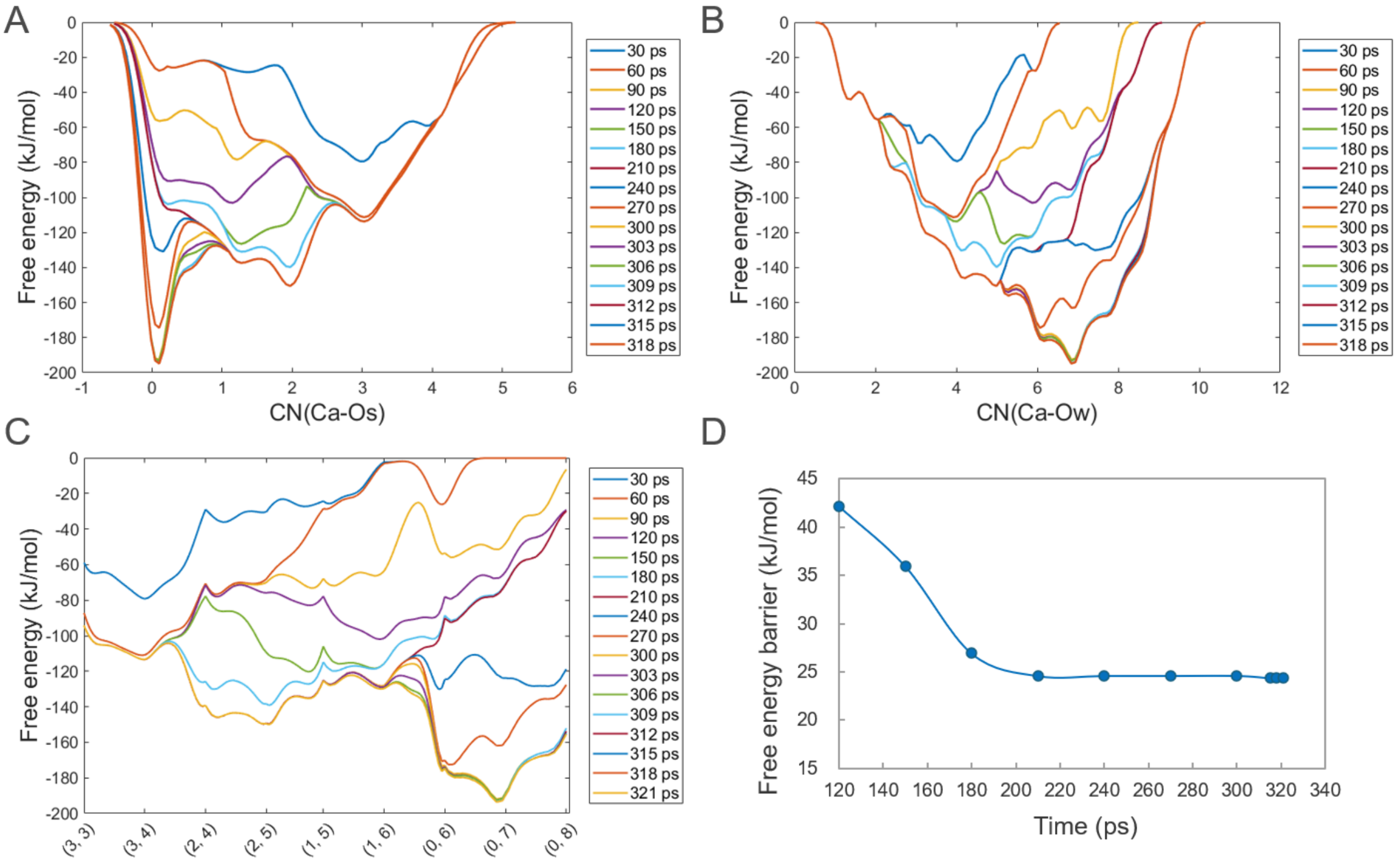

Supplementary Figure 8.

Precipitation amounts with and without considering evaporation. Evaporation was considered by linearly reducing the water volume from 0.1 mL to nearly zero (here, 0.0026 mL) over the course of 24 hours.

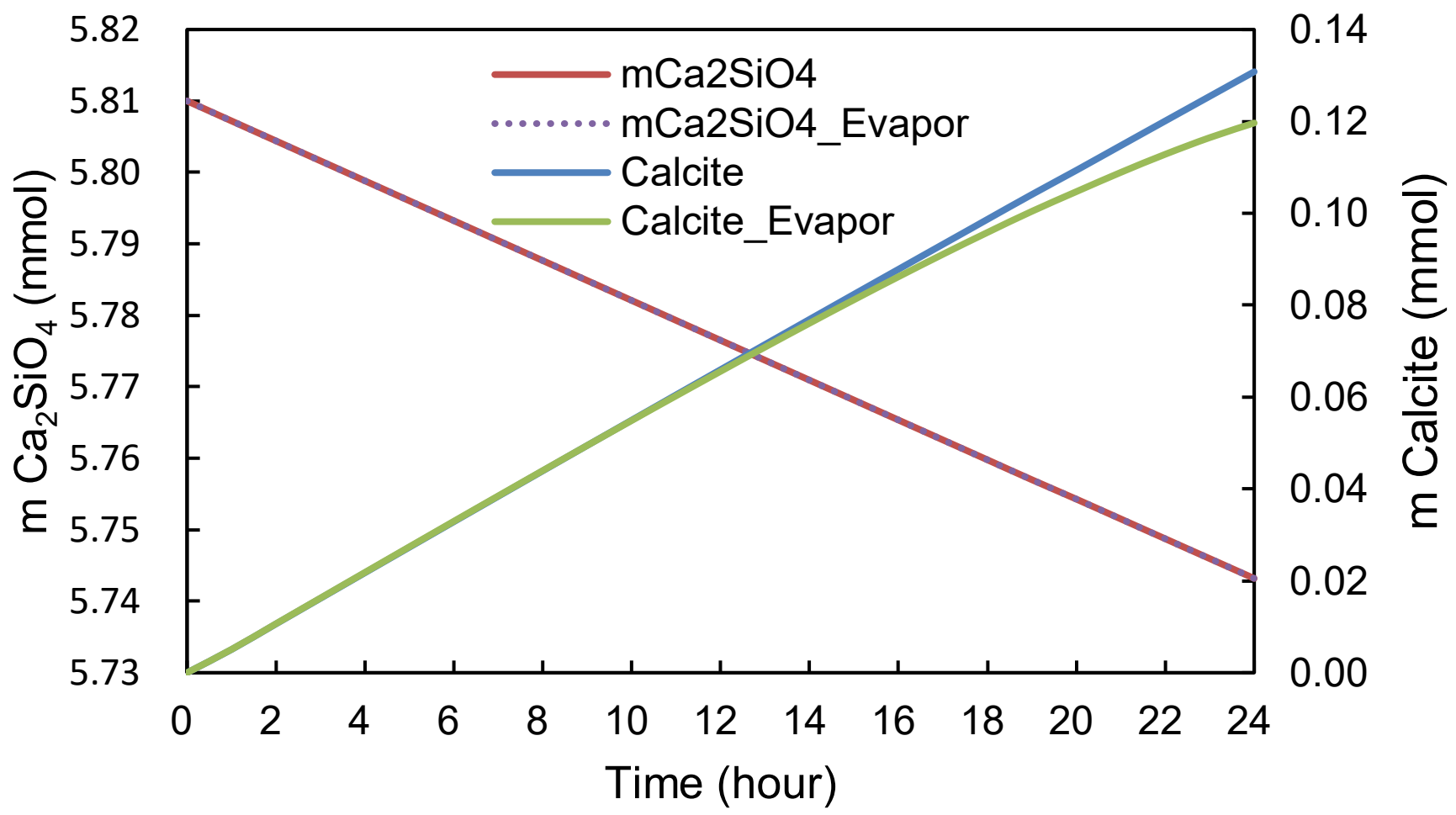

Supplementary Figure 9.

pH variations during 50 days under air, 1 atm and 2 atm CO_2 conditions.

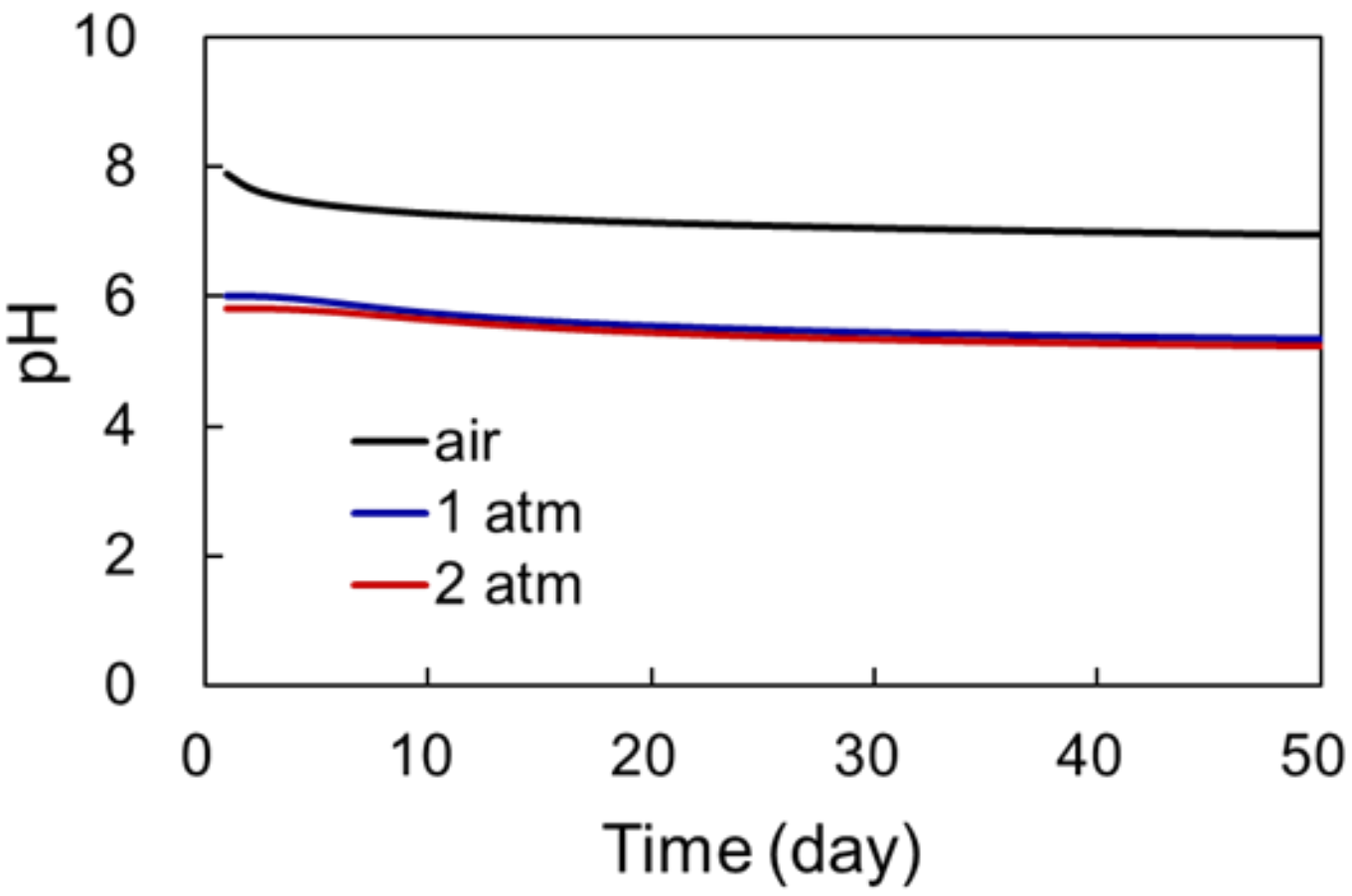

Supplementary Table 1.

The atomic volumes of Ca and Mg ions on Olivine surfaces.

Atomic Volume (Å^3)	Ca_2SiO_4	$CaMgSiO_4$	Mg_2SiO_4
M2 on surface	161	234	89
M1 in bulk	90	39	37
M2 in bulk	92	86	37

Supplementary Table 2.

Surface concentrations of initial species on Ca_2SiO_4 surface

Surface species	Surface density (number/nm^2)	Surface density (mol/m^2)
$\equiv Si{-}O^{-}\rightarrow Ca^{+}<$	5.83	9.68×10^{-6}
$>CaOH^{0}$	2.91	4.83×10^{-6}

Supplementary Table 3.

Surface reactions at the Ca_2SiO_4-water-CO_2 interface

Reaction types		Log K	Reference
1	$\equiv Si{-}O^{-}\rightarrow Ca^{+}< + H^{+} = \equiv Si{-}OH\rightarrow Ca^{+}<$	10.3	5
2	$\equiv Si{-}OH\rightarrow Ca^{+}< + H^{+} = \equiv Si{-}2OH_2^{+}\rightarrow Ca^{+}<$	3.1	5
3	$>CaOH^{0} + H^{+} = >CaOH_2^{+}$	10.0	5
4	$>CaOH^{0} = >CaO^{-} + H^{+}$	-12.1	5
5	$\equiv Si{-}O^{-}\rightarrow Ca^{+}< + CO_2 = \equiv Si{-}CO_3^{-}\rightarrow Ca^{+}<$	-1.74	ab initio
6	$\equiv Si{-}CO_3^{-} + H^{+} = \equiv Si\text{-}HCO_3^{0}$	4.90	6,7

Supplementary References

1. Wogelius, R. A. & Walther, J. V. Olivine dissolution at 25°C: Effects of pH, CO_2, and organic acids. *Geochim. Cosmochim. Acta* **55**, 943–954 (1991).
2. Casey, W. H. & Westrich, H. R. Control of dissolution rates of orthosilicate minerals by divalent metal–oxygen bonds. *Nature* **355**, 157–159 (1992).
3. Robinson, D. B., Peng, D.-Y. & Chung, S. Y.-K. The development of the Peng - Robinson equation and its application to phase equilibrium in a system containing methanol. *Fluid Phase Equilibria* **24**, 25–41 (1985).
4. Sander, R. Compilation of Henry's law constants (version 4.0) for water as solvent. *Atmospheric Chem. Phys.* **15**, 4399–4981 (2015).
5. Sverjensky, D. A. & Sahai, N. Theoretical prediction of single-site surface-protonation equilibrium constants for oxides and silicates in water. *Geochim. Cosmochim. Acta* **60**, 3773–3797 (1996).
6. Pokrovsky, O. S., Schott, J. & Thomas, F. Dolomite surface speciation and reactivity in aquatic systems. *Geochim. Cosmochim. Acta* **63**, 3133–3143 (1999).
7. Pokrovsky, O. S., Schott, J. & Thomas, F. Processes at the magnesium-bearing carbonates/solution interface. I. a surface speciation model for magnesite. *Geochim. Cosmochim. Acta* **63**, 863–880 (1999).
