# Supplementary material for: Surface carbonation governs olivine reactivity for rapid CO2 mineralization
Source: Nat Commun. 2026 Oct 14;17:11616. doi: 10.1038/s41467-026-78548-z (PMC13755434; doi:10.1038/s41467-026-78548-z)
Supplement: Supplementary file 2 [file 41467_2026_78548_MOESM3_ESM.pdf]

nature portfolio

Peer Review File

Surface carbonation governs olivine reactivity for rapid CO2 mineralization

Corresponding Author: Dr Yunfeng Liang

This file contains all reviewer reports in order by version, followed by all author rebuttals in order by version.

Version 0:

Reviewer comments:

Reviewer #1

(Remarks to the Author)
Report on the manuscript entitle "Surface carbonation governs olivine reactivity for rapid CO2 mineralization by Zihua Shao, Yunfeng Liang, Gyuhwan Jo, Keisuke Nakamura and Takeshi Tsuji submitted to Nature Communications for publication.

This paper focusses on the fundamental steps of ultramafic rocks (such as olivine in its Ca and Mg forms) carbonation process using a combination of modeling techniques namely ab initio molecular dynamics, metadynamics, and geochemical modeling. These natural crystalline solids constitute a vast global resource capable, in principle, of permanently storing all anthropogenic CO_2 through mineral carbonation. In this perspective, the submitted paper is important in the context of lowering CO_2 atmospheric concentration. Ca-olivine, produced via Ca^{2+}/Mg^{2+} exchange, nearly halves the energy demand of direct air capture, while natural olivine carbonation remains among the lowest-cost of carbon-docking strategies. Yet the large variability in olivine reactivity is not fully explained by conventional dissolution–precipitation models.

The authors show that reactivity differences arise primarily from how cations regulate nonbridging oxygen (NBO) activity. During surface carbonation, NBOs "supply" oxygen to CO_2; in Ca-olivine this lowers the Ca^{2+} dissolution barrier and accelerates ion release by more than six orders of magnitude. The resulting rate constants reproduce the experimentally observed rapid conversion of Ca-olivine under 1 atm CO_2. This multiscale framework clarifies the surface-controlled mechanisms of silicate carbonation and provides a predictive basis for large-scale CO_2 mineralization in synthetic and basaltic systems.

Overall, this is an interesting and well-written paper that can be published to NatComm when the following comments and remarks have all been adequately addressed.

First, all together, the used approach reminds me of the work of Xinping Zhu et al ["Molecular mechanisms of CO_2 mineralization on wetting nanoscale surfaces using molecular simulations and metadynamics" — Natcomm, 2025 doi: 10.1038/s41467-025-65794] on Portandite carbonation and that of Yining Gao et al ["Moisture-driven carbonation kinetics for ultrafast CO_2 mineralization", PNAS, 2025 https://doi.org/10.1073/pnas.2418239121] as these two contributions (although not ab initio but based on reactive potential), do focus on the same key surface steps that are (1) CO_2 adsorption and (2) its encounter with Ca (or Mg in the present paper) after a necessary dissolution step of the solid. But one striking difference with the present work, is the role of the interfacial water in terms of relative humidity (RH) and the formation of a nano-thick water layer at low RH that works as a nanoreactor with an optimum of CO_2 solubility. Obviously this would require a Grand Canonical approach that is simply out of range of any ab initio treatment. Instead, the authors of the present work used prescribed water thicknesses 0.7 and 1.4 nm) .

Can the authors position their work regarding to the above listed papers in terms of water content?

Second, In the work of Xinping Zhu, interfacial pH in the water film at the surface is also a key factor. pH has not been considered in the present work. Is this a strong limitation of the present paper?

Third, I do understand the power but also the limitations of the AIMD-based metadynamics in number of considered atoms and length of trajectories. In metadynamics, the "pathway" refers to the path connecting the reactant and product states as defined by the CV (or several CVs), rather than requiring the atomic positions of all atoms in the system at reactant and

product states to remain fixed throughout the simulation (as in the nudged elastic band approach as also used in the present paper). The consistency of the pathway is indicated by the CV values, which are specifically designed to capture the essential degrees of freedom relevant to the reaction or transition under study. In this context, the CV serves as the descriptor of the pathway, not the atomic coordinates. Indeed, metadynamics is an enhanced sampling method that modifies the system's energy landscape to facilitate crossing energy barriers. By design, it does not impose constraints on atomic positions but instead relies on the system's natural ability to explore various configurations within the CV-defined space, which guarantees the minimum artifacts introduced to the reaction pathway. This approach inherently accounts for atomic rearrangements, which are crucial for capturing the entropy contributions to the free energy of the system. For instance, the rearrangements of atoms, including those beyond the Ca and coordinated Ow atoms, are essential to accurately compute the free energy, particularly its entropic components.

This triggers the following regarding overall convergence. In the well-tempered metadynamics approach, one need to monitor the number of barrier re-crossings events to show that both sides of the barrier are adequately sampled. With a bias-factor applied every 30 timesteps (30 fs, that is super short), long enough simulations are required to converge the calculations. Therefore, can the authors check the convergence of their metadynamics using different criteria such as the stability of the free energy surface over time by monitoring the time evolution of the free energy surface every 1000 ??? Gaussian bias over XXX ps of simulation? This assessment is grounded in the fundamental relationship between the free energy and the accumulated bias potential, as described by: $V(s, t \rightarrow \infty) = -(1 - 1/\gamma)F(s) + C$ once the free energy surface has converged (to a constant shift C in its absolute value). If there is no observable difference between the free energy surface/profile at ZZZ ps and ZZZZ ps, this provides strong evidence of convergence in simulations. Can the authors demonstrate the convergence of their simulations along these lines?

Fourth, it is necessary to observe the height of the Gaussian bias to reduce when the free energy surface approaches convergence. Initially, the Gaussian heights are larger when the system explores new regions of the phase space. However, as the bias fills the local minima on the free energy surface, the Gaussian heights decrease. A persistently small Gaussian height indicates that no new conformational space is being explored. This behavior should be further corroborated by examining the CVs to confirm that all meaningful regions of the CV space have been sampled. Can the authors show that this is indeed the case in their simulations?

Fifth, it is essential to ensure that both sides of the free energy barrier are adequately sampled. This can be verified by observing the diffusion of CV values across a given barrier multiple times. Can the authors demonstrate this?

In summary, the authors have to put more effort in demonstrating that their well-tempered metadynamics simulations have rigorously converged in terms of configurational exploration, CV diffusion, and a stable free energy surface and barriers. These aspects are extensively discussed by Silvestri et al. [Silvestri et al., J Chem Theory Comput. 2022 Oct 11;18(10):5901-5919. doi: 10.1021/acs.jctc.2c00787]. I advise the authors to refer this metadynamics method paper.

Reviewer #2

(Remarks to the Author)
In this manuscript the authors propose the involvement of non-bridging oxygens (NBOs) in modulating the activity of olivines in mineral carbonation related reactions. The authors demonstrate through simulation the involvement of the NBOs as an oxygen source, which in combination with available water molecules and surface structure provides a highly plausible route and rationale for the large variation in reactivity seen between different mineral compositions. This addresses an important puzzle and identifies a general mechanism that is likely to be of broad interest. Although the currently included computations may turn out to be sufficient, given the many potential reactions and reaction mechanisms at mineral surfaces, this work needs to more clearly discuss the level of remaining uncertainties in explaining the reactivity variations, and more alternative scenarios that have or have not been excluded given the known limitations of the techniques applied. Beside this general request, I have one main specific concern:

1. Focusing on the theoretical methods used, the combination of ab initio molecular dynamics and metadynamics is an established and suitable choice for identifying and quantifying novel reaction pathways at surfaces. However, they remain limited in lengthscale, timescale, complexity, and fundamental accuracy. A key weakness of the present manuscript is the justification for the DFT simulation protocol – this presently uses PBE-D3 without justification. Either through finding appropriate references or additional computation, some evidence of the accuracy of this choice is needed. E.g. Reaction barriers are found to be the same as when higher-rung functionals are used. Approximations introduced at this stage could bias the observed reaction paths.

Additional minor concerns:

2. Overall the manuscript would benefit from additional editing to make the claims clearer and to include references to other pertinent literature:

2a. The abstract should be lightly edited e.g. line 19 “However, the origin of the drastic reactivity among olivine..” is incomplete and should state what is varying (?) drastically about the reactivity. Similarly, line 29 presumably refers to *previously* predicted rates.

2b. The first paragraph should contain additional citations to the recent literature to improve accessibility to non-specialists,

e.g. line 40, “With global reserves of Mg-rich silicates exceeding 105 gigatons and annual mine tailings over 400 million tons, this material offers vast and scalable potential for permanent CDR.” is unreferenced despite the plentiful literature.

2c. There are various points throughout where the authors could be more precise and include references to prior work. E.g. line 67 “This suggests the presence of alternative carbonation mechanisms not captured by current models.” (which?), line 72 “Although long considered kinetically negligible”(add reference).

3. The figures are in general well constructed, but a clearer overview figure of the surface geometry and proposed reactions considered is recommended – I do not find Fig 1F clear.

Other than the points noted above, the manuscript is in general clearly written.

Reviewer #3

(Remarks to the Author)
There are some key disconnects between this study and the natural system implications.
I appreciate that the authors are simulating systems where mineral surfaces are in contact with water, as many molecular simulation studies react CO2 with unrealistic mineral surfaces that will not be found at the surface or subsurface. However, it is not clear how these results will translates to aqueous environments, which are more abundant than the corresponding solid-water-gas surface or regolith environments.
Additionally, the authors are overstating the importance of Ca in olivine, which is only present in trace or minor amounts (<1%). The results about kinetic differences between how Ca- or Mg-rich olivine surface sites have not been shown by the authors to have a measurable impact in nature. Calculations or modeling could potentially be employed to strengthen the wider impact, but the small amount of Ca in olivine can’t be overcome in terms of what is driving the reactions. If the energy barriers and dissolution kinetics for Mg are so much lower relative to Ca, Mg reactivity remains the controlling step of olivine reactivity overall. Overall this key disconnect limits the applicability and relevance to natural systems, but it is still interesting from a surface geochemistry perspective, just for a more specialist community.
I recommend this manuscripts be updated to more realistically portray the implications for rock-CO2-water interactions, and then resubmitted to a more specialist journal focused on surface chemistry or broad mineralogy or geochemistry. A broader impact could be made if the authors also include the effects of iron, which is abundant (often 10-15%) as a cation substitute for Mg in olivine. One qualitative implication the authors may be able to suggest is that Ca may initially help olivine dissolution, as the <1% Ca in olivine tends to have a 10s-100s ppm enrichment in the rim compared to the core in natural olivine samples.

Version 1:

Reviewer comments:

Reviewer #1

(Remarks to the Author)
The revised version of the manuscript does addressed all reviewer concerns. Clearly the manuscript has gained in strength and clarity re both context and metadynamics/DFT technicalities. I would suggest the authors to explicitly cite the work of Xinping Zhu et al [“Molecular mechanisms of CO2 mineralization on wetting nanoscale surfaces using molecular simulations and metadynamics” — Natcomm, 2025 doi: 10.1038/s41467-025-65794] in the main manuscript as an outlook of their work regarding the necessity to consider pH at a future step.

Reviewer #2

(Remarks to the Author)
In this resubmission the authors have done an excellent job of addressing the questions raised by all 3 prior referees. I (reviewer 2) previously requested justification of simulation protocols, figure improvements, and some improved referencing and phrasing. In their response the authors have justified the density functional PBE-D3 choice via citations to similar studies. The methods used are up to modern standards, and I find that the revised manuscript sufficiently clearly communicates its potential weaknesses. As also noted by reviewer 1 there are numerous factors (pH, water concentrations, composition, timescale) relevant in the real-world aqueous environment. In the revised manuscript the limitations of the study and focus on a particular simplified experimental situation are more clearly indicated. The figure 1 and several key points in the text have also been improved in this revised version, e.g. the previously ambiguous abstract.

From a simulation perspective, one remaining weakness concerns reproducibility – ideally all input files and output datasets would be provided. Although sufficient high-level details are given, a complete archive would allow other practitioners to e.g. verify details of the simulation supercell sizes and lattice constants used or repeat the calculations with higher fidelity methods in future. If it is the authors’ intention to provide extensive files in the Supplemental Data, this is not clearly stated in the manuscript.

I recommend this article for publication. It provides interesting new insight into mineralization that I hope will spark follow-on research.

Reviewer #3

(Remarks to the Author)
It is apparent that the authors responded to all reviewer comments, and have improved and tuned the implications and context. This timely manuscript on an important topic related to mineral surface reactivity is ready for publication.

Open Access This Peer Review File is licensed under a Creative Commons Attribution 4.0 International License, which permits use, sharing, adaptation, distribution and reproduction in any medium or format, as long as you give appropriate credit to the original author(s) and the source, provide a link to the Creative Commons license, and indicate if changes were made.
In cases where reviewers are anonymous, credit should be given to 'Anonymous Referee' and the source.
The images or other third party material in this Peer Review File are included in the article's Creative Commons license, unless indicated otherwise in a credit line to the material. If material is not included in the article's Creative Commons license and your intended use is not permitted by statutory regulation or exceeds the permitted use, you will need to obtain permission directly from the copyright holder.
To view a copy of this license, visit https://creativecommons.org/licenses/by/4.0/

List of Responses to Reviewers' Comments and Suggestions

Dear Editor,

Thank you very much for your kind consideration and the reviewers' comments concerning our manuscript. We are pleased to see that all reviewers recognized that the results of our study are interesting. Reviewer 1 pointed out the need for more detailed metadynamics simulation convergence tests. Reviewer 2 provided comments on the use of PBE-D3 in density functional theory (DFT) simulations in our study. Reviewer 3 acknowledged the relevance of the study and suggested further discussion of implications on in situ carbon mineralization. We have carefully revised the manuscript to address the reviewers' concerns. Major revisions include:

1. To respond to **Reviewer Report 1.1, 1.2, and the general request of Reviewer 2**, we have added a discussion about the remaining uncertainties caused by factors like water conditions, pH and other possible pathways.
2. To respond to **Reviewer Report 1.3, 1.4, 1.5**, we have performed longer well-tempered metadynamics simulations and added time-dependent convergence analyses.
3. To respond to **Reviewer Report 2.1**, we added justification in DFT simulation protocols.
4. To respond to **Reviewer Report 2.3**, we improved the clarity of Fig. 1F showing the proposed reactions.
5. To respond to **Reviewer Report 3.1**, we have revised the implications on in situ carbon mineralization.

We have addressed all other minor questions (e.g., **Reviewer Report 2.2**) and improved our manuscript when appropriate. Revised parts are highlighted in blue in our revised manuscript (please see a Highlighted-in-Blue Copy). A clean copy is submitted as guided. The point-by-point responses to the reviewers' comments and changes are as follows.

Reviewer(s)' Comments to Author:

Reviewer: 1

Comments:

Report on the manuscript entitle "Surface carbonation governs olivine reactivity for rapid CO_2 mineralization by Zihua Shao, Yunfeng Liang, Gyuhwan Jo, Keisuke Nakamura and Takeshi Tsuji submitted to Nature Communications for publication.

This paper focusses on the fundamental steps of ultramafic rocks (such as olivine in its Ca and Mg forms) carbonation process using a combination of modeling techniques namely ab initio molecular dynamics, metadynamics, and geochemical modeling. These natural crystalline solids constitute a vast global resource capable, in principle, of permanently storing all anthropogenic CO_2 through mineral carbonation. In this perspective, the submitted paper is important in the context of lowering CO_2 atmospheric concentration. Ca-olivine, produced via Ca^{2+}/Mg^{2+} exchange, nearly halves the energy demand of direct air capture, while natural olivine carbonation remains among the lowest-cost of carbon-docking strategies. Yet the large variability in olivine reactivity is not fully explained by conventional dissolution–precipitation models.

The authors show that reactivity differences arise primarily from how cations regulate nonbridging oxygen (NBO) activity. During surface carbonation, NBOs "supply" oxygen to CO_2; in Ca-olivine this lowers the Ca^{2+} dissolution barrier and accelerates ion release by more than six orders of magnitude. The resulting rate constants reproduce the experimentally observed rapid conversion of Ca-olivine under 1 atm CO_2. This multiscale framework clarifies the surface-controlled mechanisms of silicate carbonation and provides a predictive basis for large-scale CO_2 mineralization in synthetic and basaltic systems.

Overall, this is an interesting and well-written paper that can be published to NatComm when the following comments and remarks have all been adequately addressed.

1.1. First, all together, the used approach reminds me of the work of Xinping Zhu et al ["Molecular mechanisms of CO_2 mineralization on wetting nanoscale surfaces using molecular simulations and metadynamics" — Natcomm, 2025 doi: 10.1038/s41467-025-65794] on Portandite carbonation and that of Yining Gao et al ["Moisture-driven carbonation kinetics for ultrafast CO_2 mineralization", PNAS, 2025 https://doi.org/10.1073/pnas.2418239121] as these two contributions (although not ab initio but based on reactive potential), do focus on the same key surface steps that are (1) CO_2 adsorption and (2) its encounter with Ca (or Mg in the present paper) after a necessary dissolution step of the solid. But one striking difference with the present work, is the role of the interfacial water in terms of relative humidity (RH) and the formation of a nano-thick water layer at low RH that works as a nanoreactor with an optimum of CO_2 solubility. Obviously this would require a Grand Canonical approach that is simply out of range of any ab initio treatment. Instead, the authors of the present work used prescribed water thicknesses 0.7 and 1.4 nm).

Can the authors position their work regarding to the above listed papers in terms of water content?

Response:

We thank the reviewer for this insightful comment. Recent studies by *Xinping Zhu et al.* [1] and *Yining Gao et al.* [2] provide important understanding of **how water content regulates mineral carbonation**. *Xinping Zhu et al.* [1] also showed that a ~1 nm water film formed at high RH provides a confined environment for CO_2 solvation, speciation, Ca dissolution, and $CaCO_3$ ion pairing. *Yining Gao et al.* [2] showed that carbonation can exhibit an optimum moisture range, while excessive water can reduce CO_2 access and shift the controlling process toward dissolution-driven carbonation.

The works of *Xinping Zhu et al.* [1] and *Yining Gao et al.* [2] primarily focus on carbonation under nanoscale or mesoscale confinement, where the relationship between relative humidity, water uptake, and nanometric water-film formation is explicitly established. In contrast, the mechanism proposed in our study is not limited to nanopores, mesopores, or a specific humidity-dependent water-film thickness. In particular, the experimental study by Chen and Kanan [*Nature 2025, 638: 972–979.*], which is used as the main benchmark for our kinetic analysis, employed powdered Ca-olivine and did not identify nanopore or mesopore confinement for the observed rapid carbonation. Our objective is therefore to describe a more general surface-carbonation mechanism in which CO_2 reacts with reactive surface oxygen sites and subsequently facilitates cation dissolution.

Regarding the experimental and geochemical modeling conditions, our PHREEQC simulations follow the experimental setting of Chen and Kanan [*Nature 2025, 638: 972–979.*], where the initial water/material ratio was 1 g/g. In their work, "*water was added to the material when it appeared dry, which corresponded to once per day.*" Based on *Yining Gao et al.*, and despite the material differences between Portland cement and Ca-olivine, a water/material ratio of 1 g/g (> 0.7 g/g) can be regarded as an aqueous condition. However, since the water/rock ratio was not maintained at a constant level in the experiments, it may have varied between 1 and 0 g/g. A water/rock ratio above 0.7 g/g corresponds to an aqueous environment, whereas it could very possibly have decreased below 0.7 g/g when the material appeared dry, corresponding to water-film conditions. Based on Xinping Zhu et al., the simulated 1.4 nm water film condition corresponds to approximately 90–95% relative humidity. In experiments under 1 atm 100% CO_2, carbonation conversion was achieved within 2 hours, possibly under an aqueous environment throughout (>0.7 g/g). In summary, experimental conditions can be regarded as an aqueous environment with a possible transition to water-film conditions.

In the AIMD simulations, the 0.7 nm water layer was chosen because this section focuses on hydrolysis and carbonation reactions with olivine. This thickness provides sufficient H_2O or CO_2 molecules for the surface to react. In the carbonation simulations, different surface water densities were considered when assessing the relative reactivity of the olivine compositions. The main purpose of these AIMD simulations was to estimate the reaction stability constants calculated by reacted ratio of NBOs and CO_2 activity, which were then incorporated into the SCM to evaluate CO_2 surface reactions under aqueous conditions. Therefore, this part does not aim to resolve the effect of water-film thickness or RH-dependent water uptake.

In the metadynamics simulations, we used a typical nanometric water-film thickness. The aim of this section was to examine whether surface carbonation can promote cation dissolution into an interfacial aqueous environment. Similar effects have also been reported in previous

studies, where carbonate species formed in interfacial water and subsequently adsorbed on mineral surfaces, reducing the Mg dissolution free energy by approximately 4 kcal/mol (~16 kJ/mol) by Ref [*J. Phys. Chem. C 2022, 126, 28: 11574–11584*]. Our previous work also observed a similar carbonate-promoted dissolution effect on pyroxene surfaces [*Shao, Zihua, et al. Environmental Science & Technology 2025, 59 (28): 14410-14418.*]. Therefore, we believe that this carbonation-induced promotion effect is not limited to our specific simulation setup.

We acknowledge that the carbonation-induced promotion of dissolution as a function of water-film thickness was not explicitly considered in this study. We agree that water condition is an important factor. In the referenced experiments, the local water condition was not strictly controlled throughout the reaction, which may introduce uncertainty into the prediction of carbonation conversion time. In particular, variations in water-film thickness could influence the carbonation promotion effect on dissolution energy. Therefore, this uncertainty may partly contribute to the remaining discrepancy between the predicted and experimental carbonation times. Future studies with more precisely controlled water/material ratios will be useful for quantifying how water-film thickness regulates surface carbonation, dissolution energetics, and overall carbonation kinetics.

We have revised the manuscript to clarify 1) how the water conditions are positioned relative to previous studies, 2) the remaining uncertainties associated with water conditions, and 3) the need to consider water conditions as an influencing factor on dissolution energetics in future work.

Action:

We have added the details in the “Simulation models” section as follows:

This 0.7 nm interfacial layer was chosen to provide sufficient H_2O or CO_2 molecules for hydrolysis and carbonation reactions within the simulation cell. In the carbonation simulations, different surface water densities were considered when assessing the relative reactivity of the olivine compositions.

We have added the details in the “Discussion” section as follows:

The role of CO_2 surface reactions may be underestimated if they are considered only under very water-limited conditions. Under extremely low water content, CO_2 surface reactions may occur on mineral surfaces, but the subsequent dissolution and precipitation processes are limited. As a result, their contribution to measurable carbonation conversion can be difficult to detect experimentally and considered as minimal. In contrast, we propose that under wet or partially wet conditions, CO_2 surface reactions can proceed continuously together with the dissolution–precipitation process. By reducing the dissolution barrier and enhancing the apparent dissolution rate as a function of CO_2 concentration, surface carbonation can effectively accelerate cation release, indicating that CO_2 surface reactions serve as an important additional mechanism for rapid carbon mineralization. The local water condition in the referenced experiments was not strictly controlled throughout the reaction, because water was added when the material appeared dry. Therefore, the system may have transitioned between aqueous and water-film conditions (< 0.7 g/g) [31] during carbonation. This introduces uncertainty into the prediction of carbonation conversion time, because water-film thickness can influence metal ion dissolution and the carbonation-induced reduction in dissolution free

energy. Such sensitivity is consistent with previous demonstrations that pathway-based ion-release free energies at solid–water interfaces depend on the definition of the solution state and the configurational volume sampled by the detached ion [23]. Future studies combining controlled water/material-ratio experiments and simulations will be needed to quantitatively connect water-film thickness, dissolution energetics, and olivine carbonation kinetics.

1.2. Second, In the work of Xinping Zhu, interfacial pH in the water film at the surface is also a key factor. pH has not been considered in the present work. Is this a strong limitation of the present paper?

Response:

We thank the reviewer for raising this important point. The pH environment of nanometric water films can strongly influence mineral carbonation, as demonstrated by Xinping Zhu et al. for portlandite, where the water-film pH modulates Ca dissolution, CO_2 speciation, and $CaCO_3$ ion pairing.

In the first section "surface reactivity of olivine with CO_2", we did not explicitly vary the interfacial pH when examining the CO_2 surface reaction. This is because surface reactions, especially the direct reaction between CO_2 and surface NBOs to form surface carbonate species, do not involve H^+ as an explicit reactant in the elementary step. The AIMD calculations were used to calculate the stability constant of surface reactions at NBOs based on the reaction ratio of NBOs and activity of CO_2. The influence of pH on CO_2 availability (ratio of CO_2 molecules) was subsequently considered in the surface complexation modeling.

In the second section "surface carbonation facilitates cation dissolution", we agree that further clarification is needed. The MTD simulations were used to quantify the promotion effect of surface carbonation on Ca dissolution under a neutral condition, rather than to resolve the pH dependence of this barrier reduction. We have clarified this point in the revised manuscript. Future work that explicitly varies the protonation state or local pH of confined water films would be valuable for further quantifying how interfacial pH modulates this carbonation-induced enhancement.

In the third section "CO_2 uptake kinetics under ambient conditions", the effects of pH on surface speciation, carbonate speciation, and dissolution kinetics were considered in the surface complexation modeling (SCM) and geochemical modeling. Specifically, the SCM includes protonation and deprotonation reactions of surface sites, as well as the conversion between surface carbonate and bicarbonate species. It also accounts for the effect of pH on dissolved carbon species distributions, including the relative abundance of molecular CO_2. In addition, the pH-dependent dissolution rate was included in the geochemical model. Under air-equilibrated conditions, the solution pH is approximately neutral, whereas under 1 atm CO_2 the pH decreases to about 5.7–5.1, leading to an approximately 0.33 log-unit increase in the olivine dissolution rate. The PHREEQC-based dissolution and precipitation simulations therefore include the effect of pH on dissolution rate, as described in Supplementary Note 1. However, the carbonation-induced enhancement factor for cation dissolution was treated as a constant over these neutral to near-neutral conditions.

In summary, the main simplification in the present approach is that the carbonation-induced promotion of Ca dissolution was quantified by MTD under a neutral condition. We have now clarified this point in the revised manuscript and added that the explicit effect of interfacial

pH on this promotion effect is an important direction for future work.

Action:

We have added the clarification in the "Surface carbonation facilitates cation dissolution" section as follows:

It should be noted that the carbonation-induced reduction in the Ca dissolution barrier under a neutral condition and applied in the next section for neutral or close to neutral conditions, while future simulations with controlled protonation states would be useful for resolving the additional influence of local interfacial pH.

1.3. Third, I do understand the power but also the limitations of the AIMD-based metadynamics in number of considered atoms and length of trajectories. In metadynamics, the "pathway" refers to the path connecting the reactant and product states as defined by the CV (or several CVs), rather than requiring the atomic positions of all atoms in the system at reactant and product states to remain fixed throughout the simulation (as in the nudged elastic band approach as also used in the present paper). The consistency of the pathway is indicated by the CV values, which are specifically designed to capture the essential degrees of freedom relevant to the reaction or transition under study. In this context, the CV serves as the descriptor of the pathway, not the atomic coordinates. Indeed, metadynamics is an enhanced sampling method that modifies the system's energy landscape to facilitate crossing energy barriers. By design, it does not impose constraints on atomic positions but instead relies on the system's natural ability to explore various configurations within the CV-defined space, which guarantees the minimum artifacts introduced to the reaction pathway. This approach inherently accounts for atomic rearrangements, which are crucial for capturing the entropy contributions to the free energy of the system. For instance, the rearrangements of atoms, including those beyond the Ca and coordinated Ow atoms, are essential to accurately compute the free energy, particularly its entropic components.

This triggers the following regarding overall convergence. In the well-tempered metadynamics approach, one needs to monitor the number of barrier re-crossings events to show that both sides of the barrier are adequately sampled. With a bias-factor applied every 30 timesteps (30 fs, that is super short), long enough simulations are required to converge the calculations. Therefore, can the authors check the convergence of their metadynamics using different criteria such as the stability of the free energy surface over time by monitoring the time evolution of the free energy surface every 1000 ??? Gaussian bias over XXX ps of simulation? This assessment is grounded in the fundamental relationship between the free energy and the accumulated bias potential, as described by: $V(s, t \rightarrow \infty) = -(1 - 1/\gamma)F(s) + C$ ***once the free energy surface has converged (to a constant shift C in its absolute value). If there is no observable difference between the free energy surface/profile at ZZZ ps and ZZZZ ps, this provides strong evidence of convergence in simulations. Can the authors demonstrate the convergence of their simulations along these lines?***

Response:

We thank the reviewer for emphasizing the importance of evaluating the time-dependent convergence of the well-tempered metadynamics simulations.

Similar metadynamics settings have been used in previous ab initio studies of ion dissolution from mineral surfaces. For example,
[1] Li, Yunjian, et al. Ab initio mechanism revealing for tricalcium silicate dissolution. Nature Communications 2022, 13(1): 1253.
[2] Chen, H. Replacement reactions for carbon geosequestration may be faster in calcium olivine vs magnesium olivine. Communications Earth & Environment 2023, 4(1): 359.

In Ref [1], Gaussian hills were deposited every 30 timesteps (timestep = 1 fs) with an initial height of 3.5 kJ/mol. In Ref [2], Gaussian hills were deposited every 30 timesteps (timestep = 1 fs) with an initial height of 2.5 kJ/mol. We have also added the citations of these two references in the method section in the revised manuscript.

To address this point, we have performed longer well-tempered metadynamics simulations and added new convergence analyses for both uncarbonated and carbonated Ca dissolution. For the uncarbonated Ca system, the free energy surface was reconstructed every 3 ps, corresponding to every 100 deposited Gaussian kernels. The one-dimensional free-energy profiles along CN(Ca–Os), CN(Ca–Ow), and the projected dissolution pathway were compared throughout the simulation.

As shown in the newly added Fig. S4, the free-energy profile evolves significantly during the early stage as the bias fills the initial basin but becomes stable in the later stage. In particular, the rate-limiting free-energy barrier approaches a plateau after sufficient sampling, indicating that the dominant dissolution pathway and barrier are no longer sensitive to additional bias deposition.

For the carbonated Ca system, a longer well-tempered metadynamics simulation was performed until convergence. The free-energy surface was monitored every 30 ps during the first 300 ps and every 3 ps during the final 18 ps, as shown in Fig. S6. This analysis shows that the broad features of the free-energy surface are established, while the final-stage profiles remain nearly unchanged within the sampled pathway. The rate-limiting barrier also reaches a stable value, supporting the convergence of the carbonated Ca dissolution free-energy profile.

These additional analyses demonstrate that the reported free-energy barriers are not extracted from a single transient crossing event, but from time-dependent free-energy surfaces that become stable within the CV space. We have therefore revised the supporting information to include the time evolution of the free-energy profiles and barrier variations for both systems.

The updated dissolution barrier difference has also been incorporated into Figs. 2 and 3. Based on the extended metadynamics simulations and the additional convergence tests, Figs. 2A and 2B have been revised. Using the updated barrier difference, we further updated Fig. 3E to include a sensitivity analysis of the barrier difference.

Action:

We have added Fig. S5 and Fig. S7 to show the time-dependent convergence of the free-energy surfaces and free-energy barriers:

Supplementary Figure 5.
Convergence tests for well-tempered metadynamics simulation of uncarbonated Ca dissolution.

(A, B) The free energy surface as a function of the CN(Ca-Os) or CN(Ca-Ow) every 3 ps (100 Gaussian kernels deposited) along the simulation time. (C) The free energy surface along the dissolution pathway every 3 ps. (D) Free energy barrier variations along the simulation time.

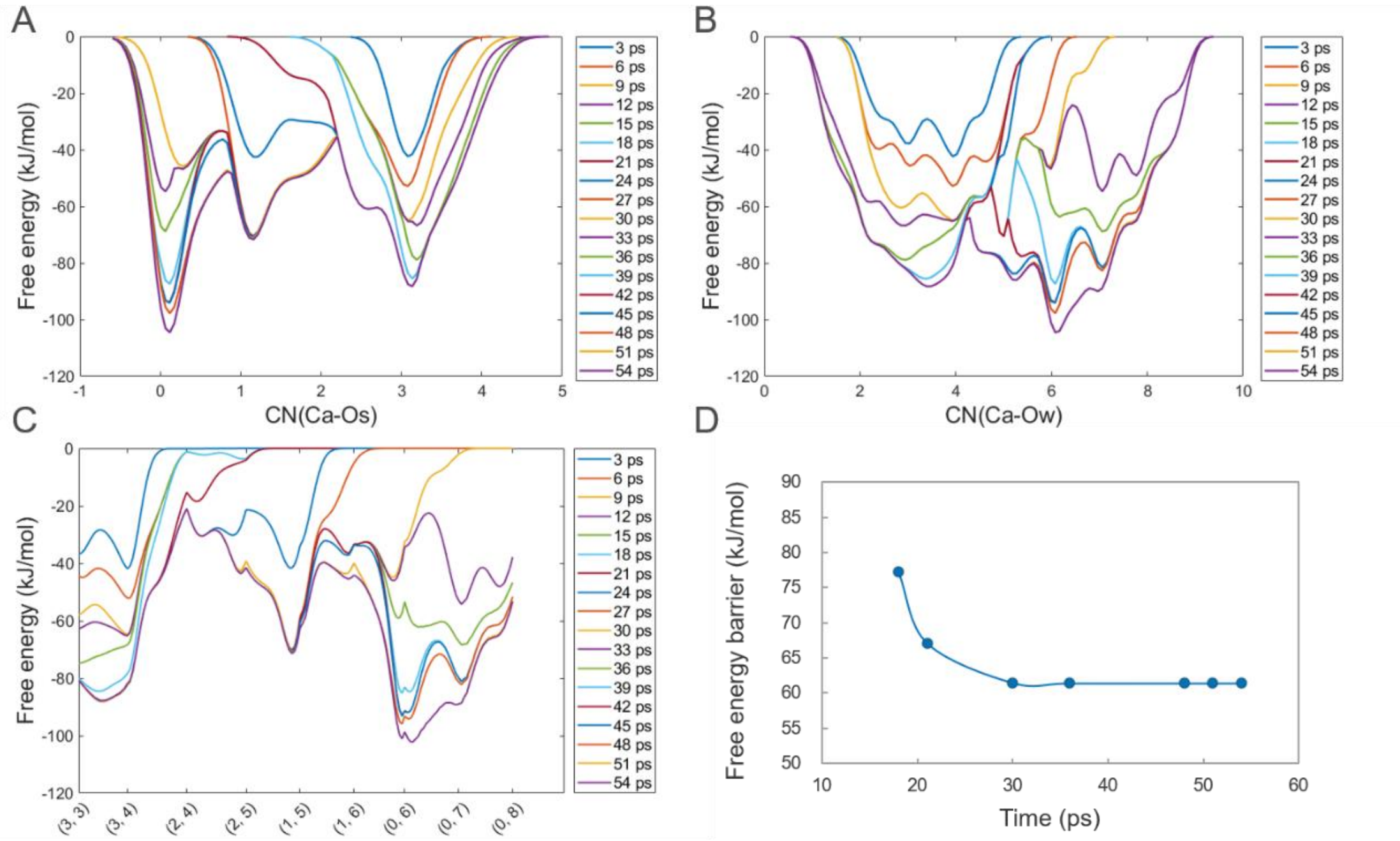

Supplementary Figure 7.

Convergence tests for well-tempered metadynamics simulation of carbonated Ca dissolution. (A, B) The free energy surface as a function of the CN(Ca-Os) or CN(Ca-Ow) every 30 ps during the first 300 ps and every 3 ps (100 Gaussian kernels deposited) during the last 18 ps. (C) The free energy surface along the dissolution pathway along the simulation time. (D) Free energy barrier variations along the simulation time.

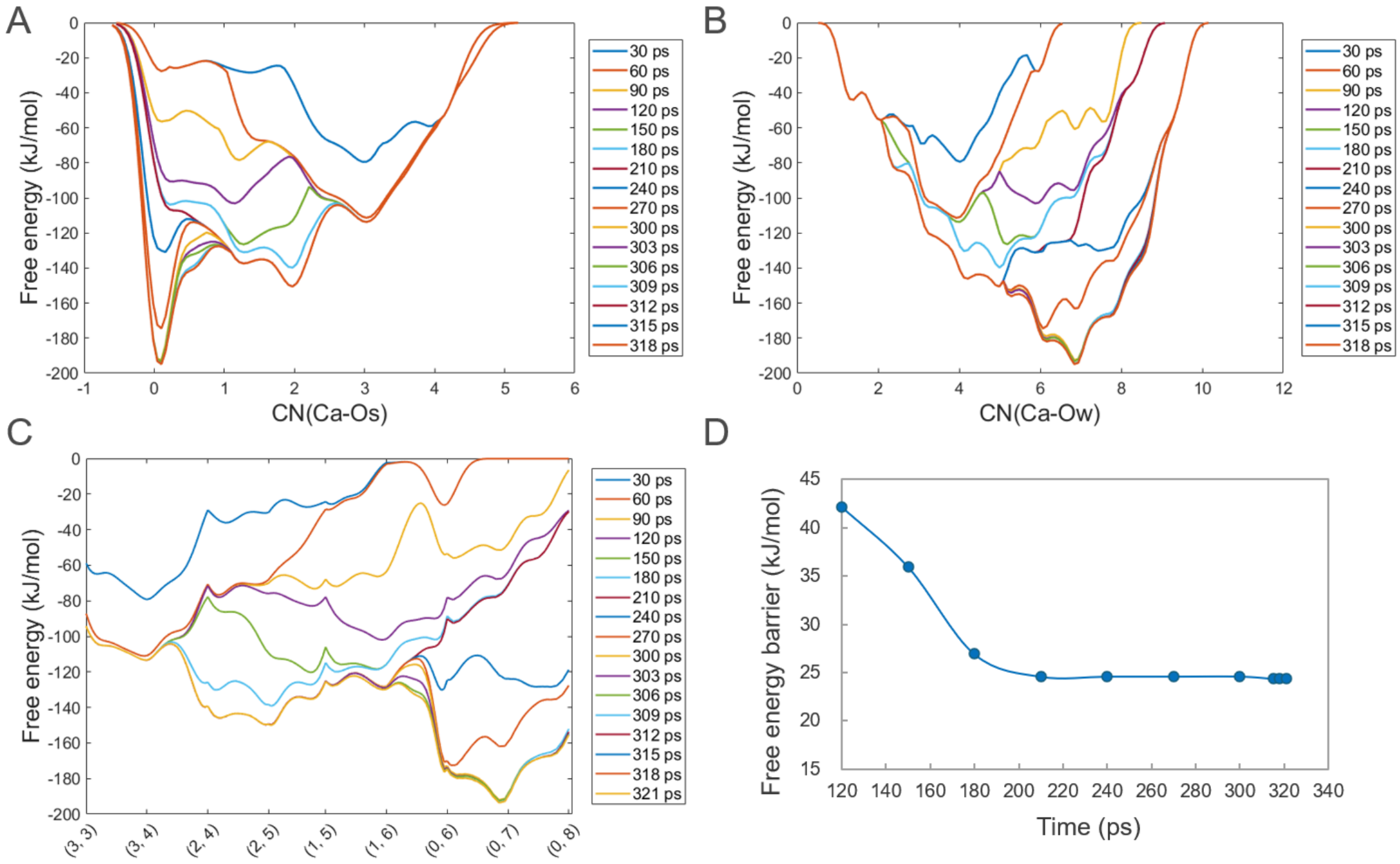

We have modified Fig. 2 (A and B) and Fig. 3 C (right):

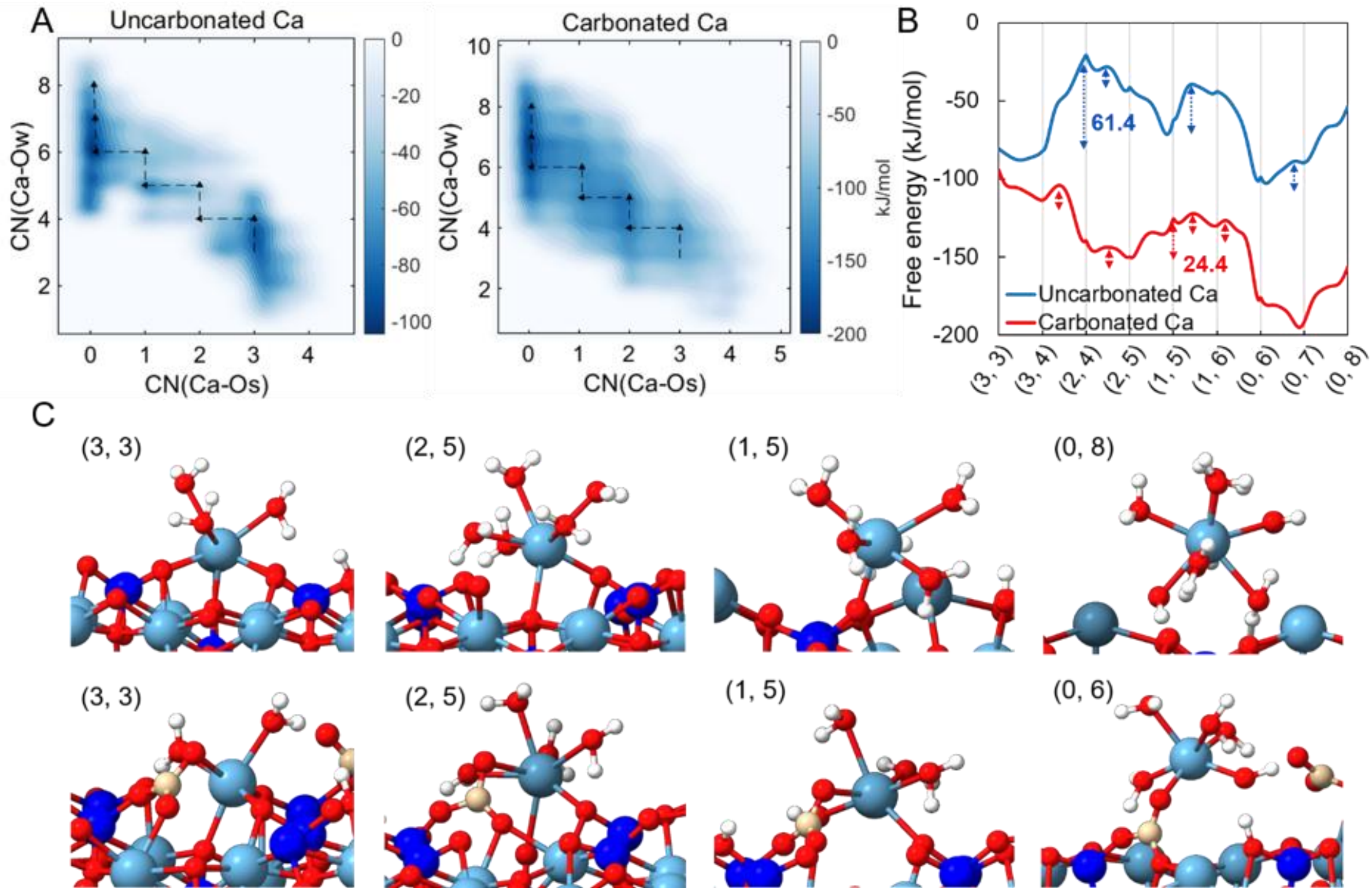

Fig. 2. Dissolution behavior of uncarbonated and carbonated Ca in olivine. (A) Free energy surface of uncarbonated Ca dissolution. The black dashed line indicates the dissolution pathway. CN(Ca–Os) denotes the coordination number between Ca and silicate oxygen, while CN(Ca–Ow) denotes the coordination number between Ca and water oxygen. (B) Free energy evolutions of uncarbonated and carbonated Ca ions. (C) Snapshots of uncarbonated and carbonated Ca on Ca_2SiO_4 surface. Calcium, silicon, oxygen, carbon, and hydrogen atoms are shown in light blue, dark blue, red, brown, and white respectively. The energy barrier is reduced from 61.4 kJ/mol (uncarbonated) to 24.4 kJ/mol (carbonated), indicating that surface carbonation substantially accelerates Ca^{2+} dissolution. Based on transition state theory, this reduction leads to a dissolution rate increase of ~ 3.0 × 10^6-fold.

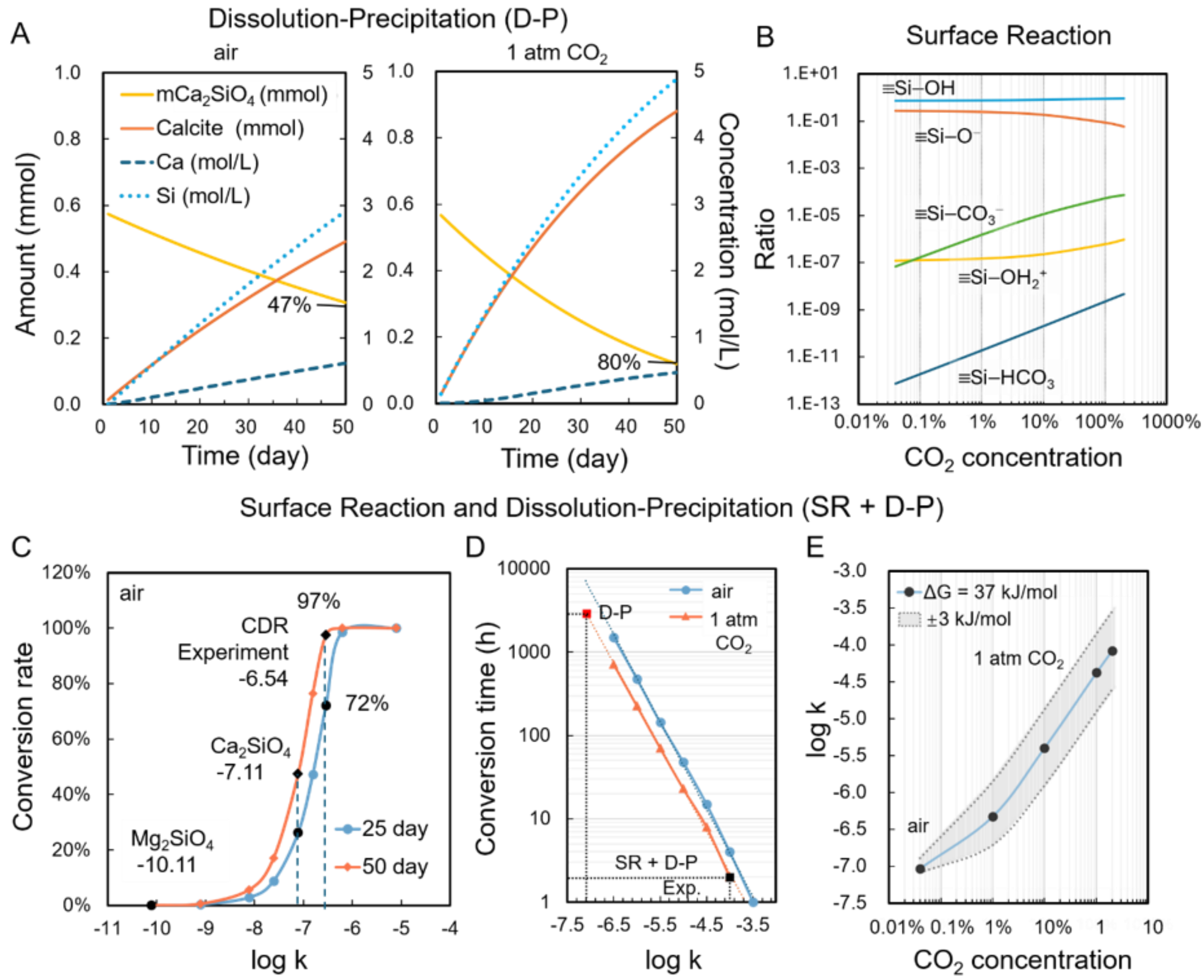

Fig. 3. Carbonation kinetics of Ca-olivine governed by coupled dissolution-precipitation (D-P) and surface reactions (SR). (A) Time-dependent dissolution of Ca-olivine (Ca_2SiO_4) and calcite precipitation under air and 1 atm CO_2. (B) Ratio of surface species of Ca_2SiO_4 with increasing CO_2 concentration, showing the transition from ≡Si–O⁻ to ≡Si–CO_3^- and ≡Si–HCO_3. CO_2 concentration is expressed relative to the partial pressure of CO_2 in air (0.04 % at 1 atm). Thus, 100 % CO_2 corresponds to 1 atm pCO_2, and 200 % CO_2 represents 2 atm CO_2. (C) Dependence of carbonation rate and time on rate constant (k) and CO_2 concentration. Conversion rate of olivine as a function of dissolution rate constant k. (D) Conversion time of olivine as a function of dissolution rate constant k. The red and black squares represent the conversion times predicted by the D–P and combined SR + D–P mechanisms, respectively. The latter overlaps with the experimental conversion time of 2 h (Exp.). (E) The relationship between apparent dissolution rate constant k and CO_2 concentration. The gray area indicated the apparent dissolution rate change by ± 3 kJ/mol (from 34.0 kJ/mol to 40.0 kJ/mol). The figure shows that dissolution-precipitation mechanism alone cannot reproduce the experiment. By considering the CO_2 surface reaction, the apparent dissolution rate increases with log k' increasing from -7.11 to –4.08, and the estimated conversion time is significantly reduced. This reproduces experimental order of magnitude well, thereby helping to resolve the long-standing mismatch with experiments.

1.4. Fourth, it is necessary to observe the height of the Gaussian bias to reduce when the free energy surface approaches convergence. Initially, the Gaussian heights are larger when the system explores new regions of the phase space. However, as the bias fills the local minima on the free energy surface, the Gaussian heights decrease. A persistently

small Gaussian height indicates that no new conformational space is being explored. This behavior should be further corroborated by examining the CVs to confirm that all meaningful regions of the CV space have been sampled. Can the authors show that this is indeed the case in their simulations?

Response:

We thank the reviewer for this helpful suggestion. We have revised the supporting information to include these analyses in Figs. S4 and S6. Fig. S4 shows the time evolution of CN(Ca–Os), CN(Ca–Ow), and Gaussian hill height for uncarbonated Ca dissolution, while Fig. S6 provides the corresponding analysis for carbonated Ca dissolution. These results are further supported by the time-dependent free-energy profiles and barrier variations shown in Figs. S5 and S7.

For the uncarbonated Ca system, CN(Ca–Os) decreases while CN(Ca–Ow) increases during the simulation, indicating the transition from a surface-bound Ca state to a water-coordinated Ca state. During this process, the Gaussian hill height decreases from its initial value and remains relatively small in the later stage of the simulation (50 ps). For the carbonated Ca system, a similar trend is observed. The Gaussian hill height decreases close to the end of the simulation. The simultaneous observation of CV returning to the initial state and reduced Gaussian hill height therefore supports the convergence of the well-tempered metadynamics simulations.

Action:

We have added Fig. S4 and Fig. S6:
Supplementary Figure 4.
Time evolution of CN(Ca-Os), CN(Ca-Ow) and Gaussian hill height during the well-tempered metadynamics simulation for uncarbonated Ca dissolution.

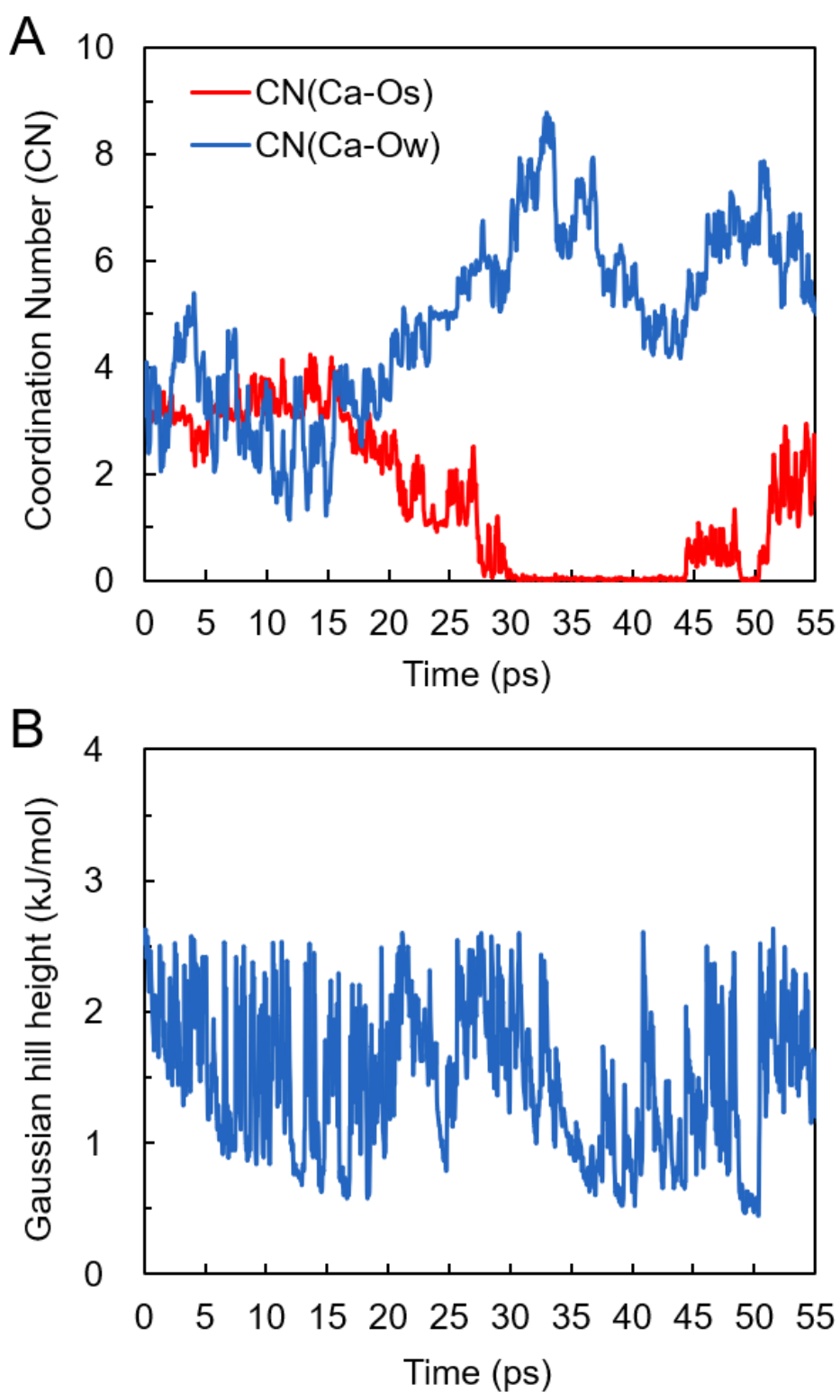

Supplementary Figure 6.
Time evolution of CN(Ca-Os), CN(Ca-Ow) (A) and Gaussian hill height (B) during the well-tempered metadynamics simulation for carbonated Ca dissolution.

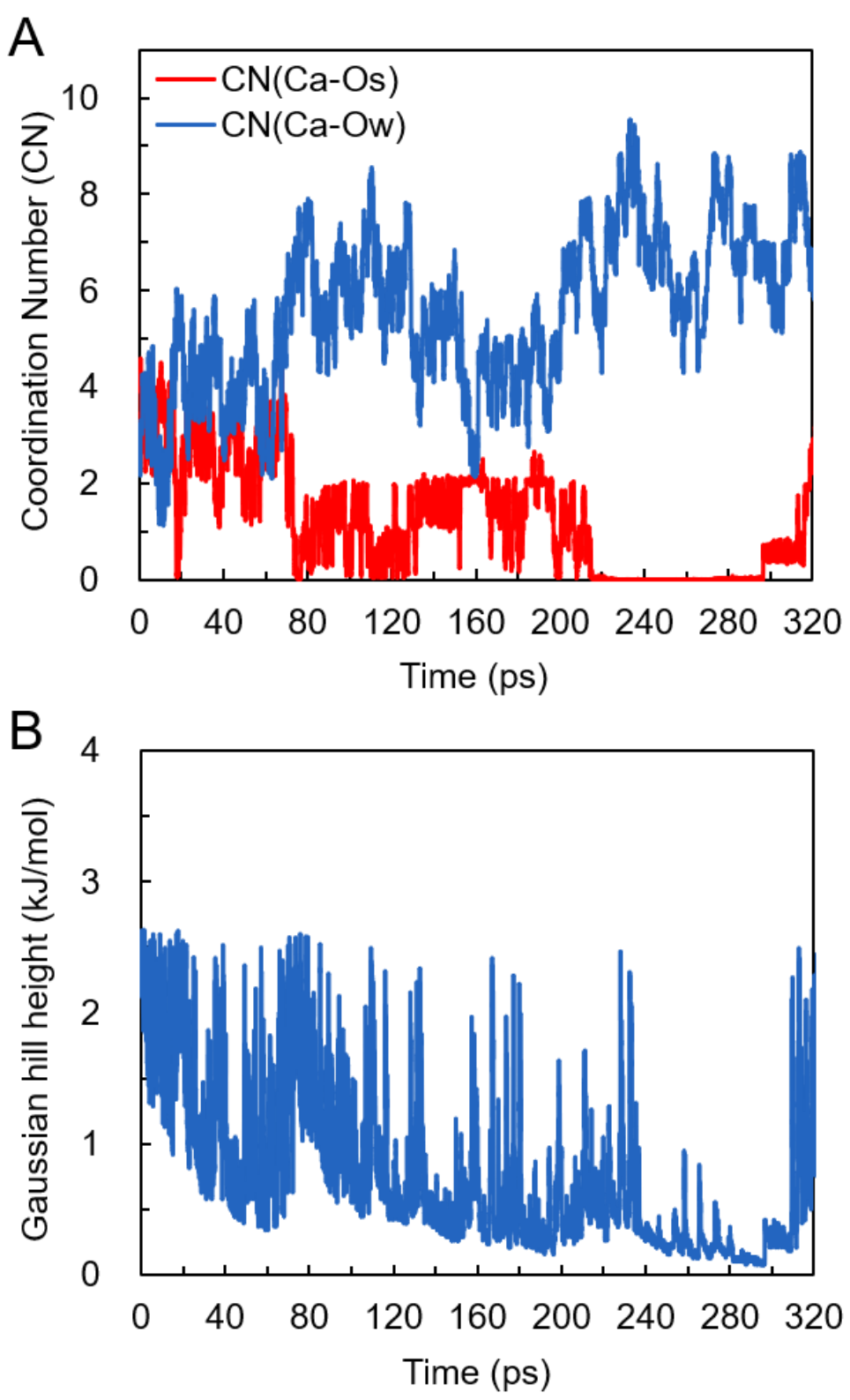

1.5. Fifth, it is essential to ensure that both sides of the free energy barrier are adequately sampled. This can be verified by observing the diffusion of CV values across a given barrier multiple times. Can the authors demonstrate this?

In summary, the authors have to put more effort in demonstrating that their well-tempered metadynamics simulations have rigorously converged in terms of configurational exploration, CV diffusion, and a stable free energy surface and barriers. These aspects are extensively discussed by Silvestri et al. [Silvestri et al., J Chem Theory Comput. 2022 Oct 11;18(10):5901-5919. doi: 10.1021/acs.jctc.2c00787]. I advise the authors to refer this metadynamics method paper.

Response:

We thank the reviewer for this important comment.

The time evolution of the CVs shows that both sides of the relevant dissolution barrier were sampled. For the uncarbonated Ca system, the CV trajectory evolves from the initial surface-bound state to the water-coordinated state, covering the intermediate coordination states along the dissolution pathway. For the carbonated Ca system, the CV trajectory also shows the loss of Ca–surface oxygen coordination and the formation of Ca coordination with water/carbonate oxygens. In both systems, the simulations did not remain confined to the initial reactant basin but crossed the barrier region as shown in Fig. S4 and S6.

Similar CV behavior has been observed in the AIMD-based metadynamics study by [*Li, Yunxian et al. Nature Communications 2022, 13(1): 1253*] on tricalcium silicate dissolution, where CN(Ca–Os) and CN(Ca–Ow) also show fluctuating evolution.

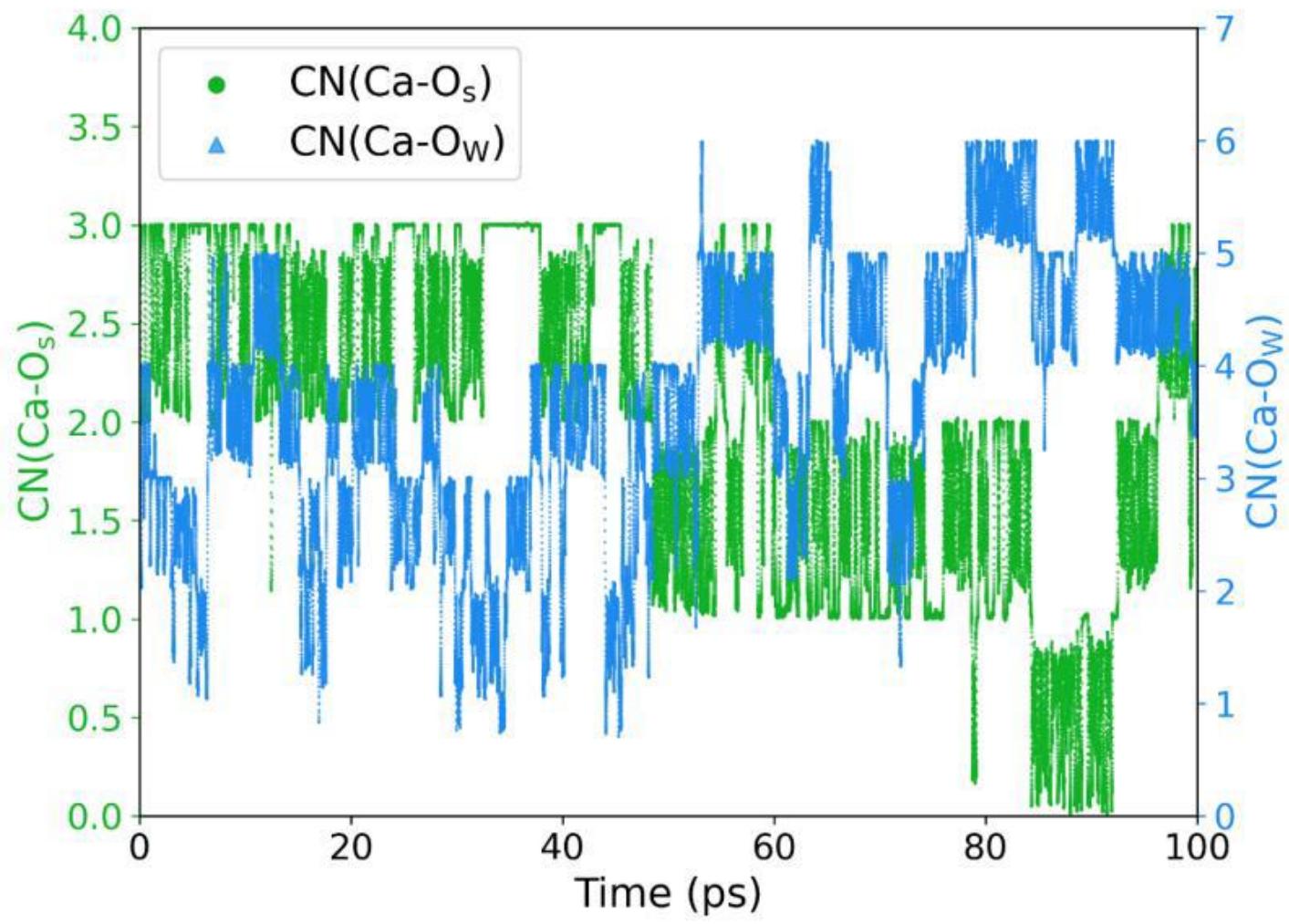

Supplementary Figure 2. Time evolution of CN(Ca-Os) and CN(Ca-Ow) during the whole simulation time of the well-tempered metadynamics simulations for dissolution of Caα.

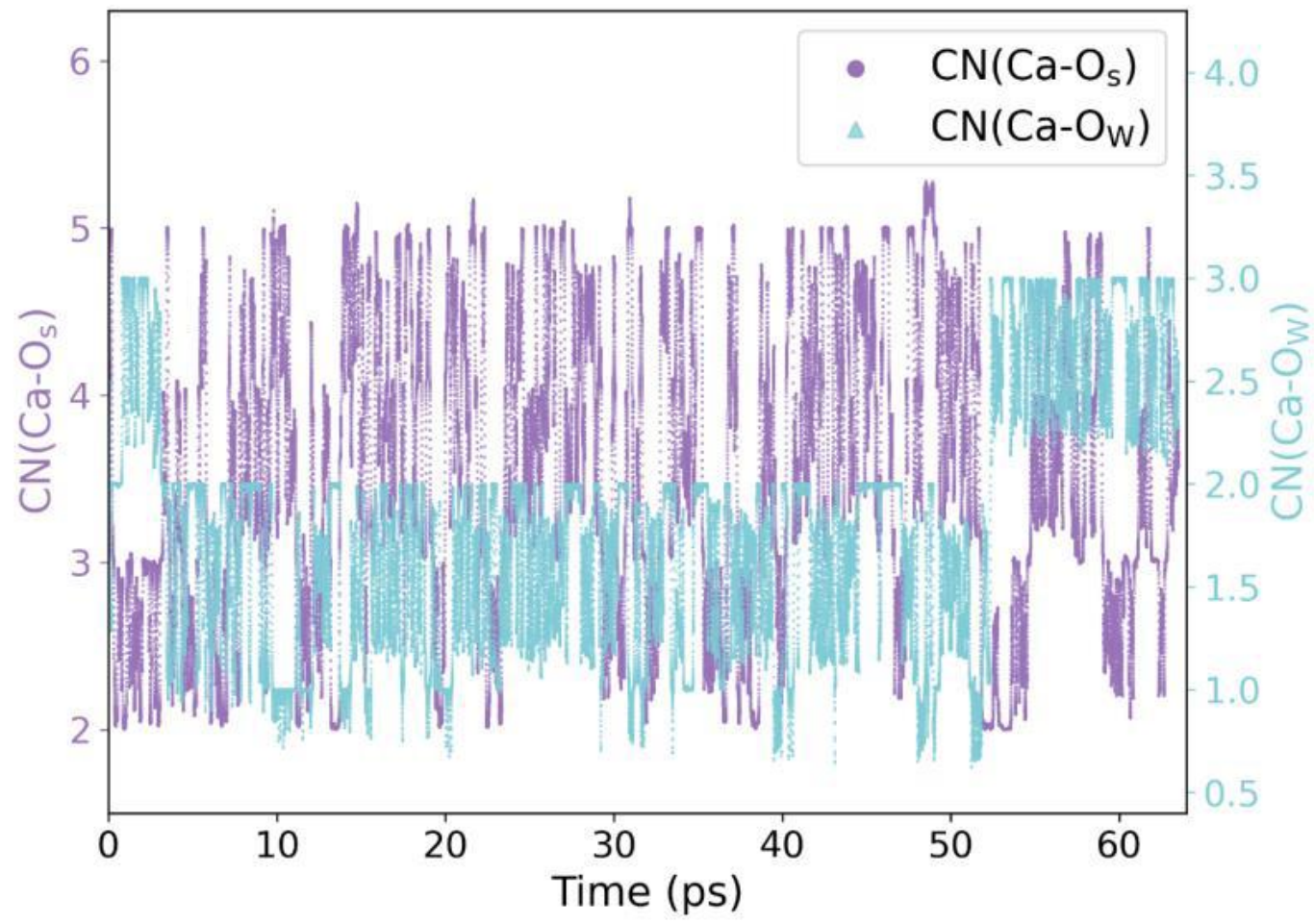

Supplementary Figure 4. Time evolution of CN(Ca-Os) and CN(Ca-Ow) during the whole simulation time of the well-tempered metadynamics simulations for dissolution of Caβ with CN(Ca-Os) from 5 to 2.

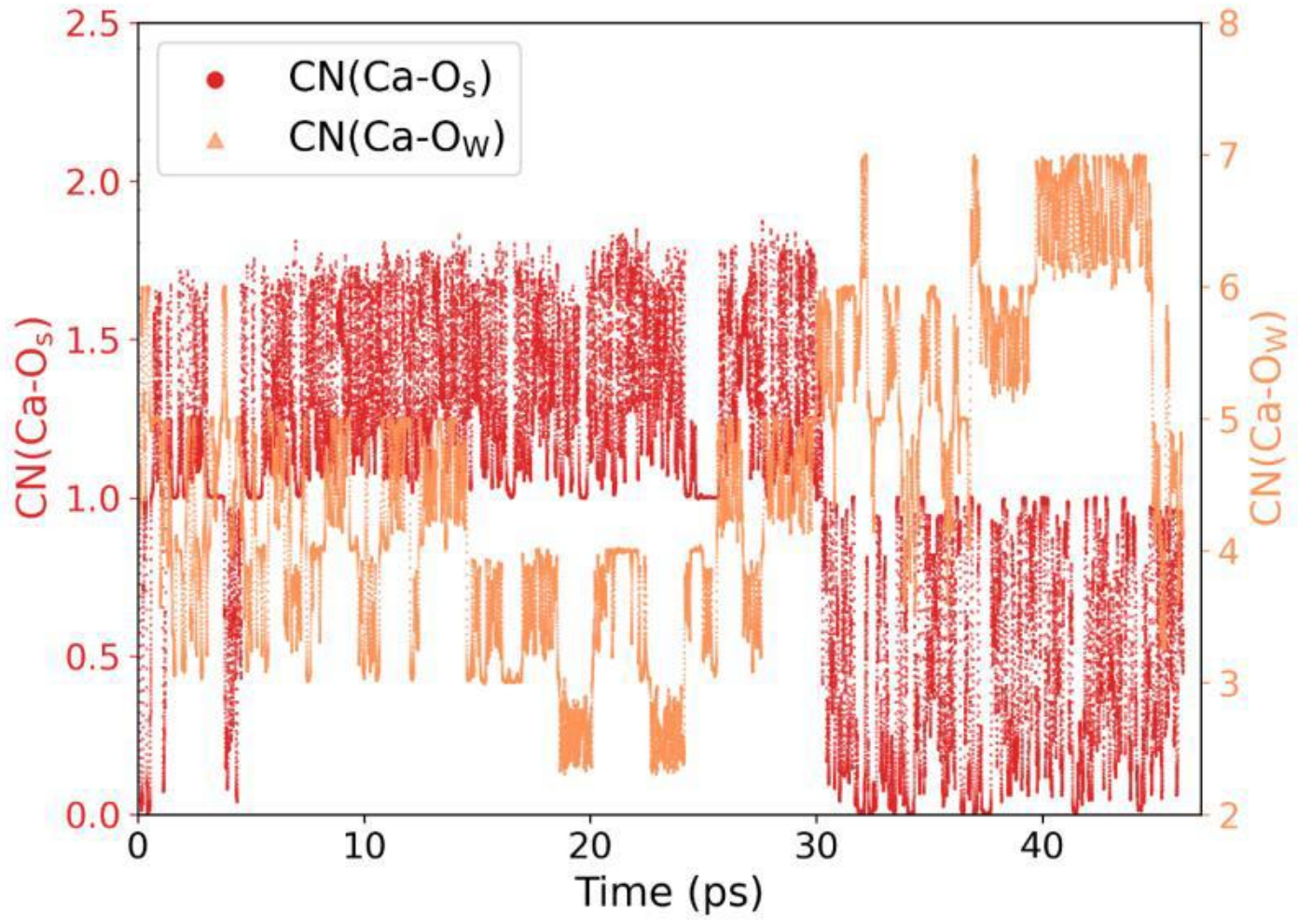

Supplementary Figure 6. Time evolution of CN(Ca-Os) and CN(Ca-Ow) during the whole simulation time of the well-tempered metadynamics simulations for dissolution of Caβ with CN(Ca-Os) from 2 to 0.

We also appreciate the reviewer for recommending the relevant work by Silvestri et al.

Action:

We have cited Silvestri et al. (Ref. 23) in the revised manuscript for metadynamics.

In "Introduction" section:
More broadly, pathway-based free-energy methods such as metadynamics have been shown to provide mechanistic insight into ion detachment from solid–water interfaces [23].

In the "Surface carbonation facilitates cation dissolution" section:

In this analysis, the MTD-derived free-energy profiles are used primarily to compare relative

detachment barriers under consistent simulation settings, rather than to determine absolute standard-state dissolution free energies [23].

In the “Discussion” section:

Following previous work on ion removal from solid–water interfaces, we interpret these pathway-based free-energy profiles as mechanistic descriptors of relative detachment barriers and associated kinetic changes [23].

Such sensitivity is consistent with previous demonstrations that pathway-based ion-release free energies at solid–water interfaces depend on the definition of the solution state and the configurational volume sampled by the detached ion [23].

In the “Method/Metadynamics” section:

These metadynamics parameters were selected following previous applications of well-tempered metadynamics to ion dissolution from mineral surfaces and solid–water interfaces [22,23,39].

Reviewer: 2

Comments:

In this manuscript the authors propose the involvement of non-bridging oxygens (NBOs) in modulating the activity of olivines in mineral carbonation related reactions. The authors demonstrate through simulation the involvement of the NBOs as an oxygen source, which in combination with available water molecules and surface structure provides a highly plausible route and rationale for the large variation in reactivity seen between different mineral compositions. This addresses an important puzzle and identifies a general mechanism that is likely to be of broad interest. Although the currently included computations may turn out to be sufficient, given the many potential reactions and reaction mechanisms at mineral surfaces, this work needs to more clearly discuss the level of remaining uncertainties in explaining the reactivity variations, and more alternative scenarios that have or have not been excluded given the known limitations of the techniques applied.

Response:

Thank you for this valuable comment. We have added several discussions about the remaining uncertainty.

Action:

In the section "CO_2 uptake kinetics under ambient conditions," we have added a sensitivity analysis examining how the predicted carbonation rate varies with the free-energy barrier, thereby illustrating the uncertainty associated with changes in the barrier.

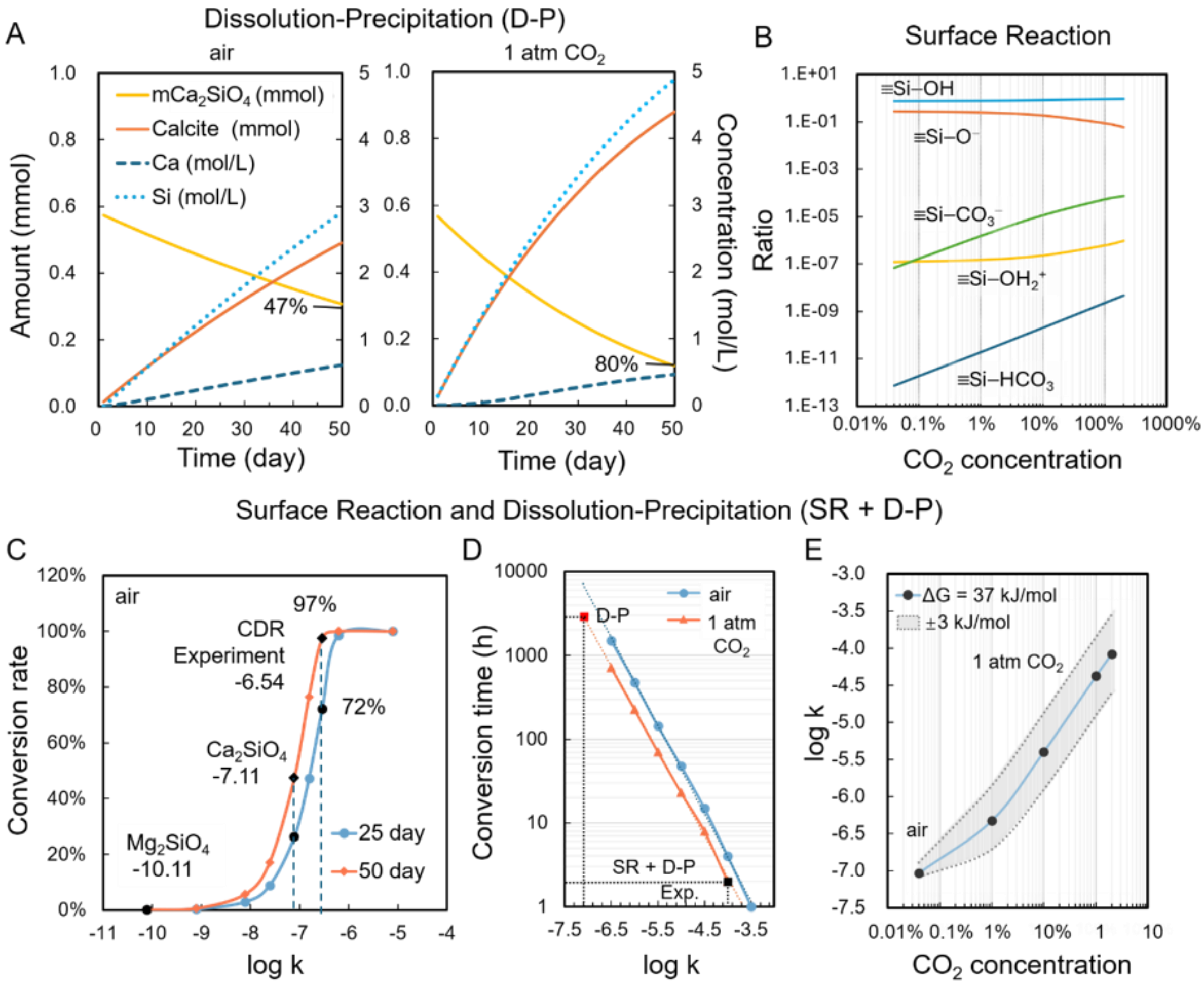

Fig. 3. Carbonation kinetics of Ca-olivine governed by coupled dissolution-precipitation (D-P) and surface reactions (SR). (A) Time-dependent dissolution of Ca-olivine (Ca_2SiO_4) and calcite precipitation under air and 1 atm CO_2. (B) Ratio of surface species of Ca_2SiO_4 with increasing CO_2 concentration, showing the transition from ≡Si–O⁻ to $\equiv Si–CO_3^-$ and $\equiv Si–HCO_3$. CO_2 concentration is expressed relative to the partial pressure of CO_2 in air (0.04 % at 1 atm). Thus, 100 % CO_2 corresponds to 1 atm pCO_2, and 200 % CO_2 represents 2 atm CO_2. (C) Dependence of carbonation rate and time on rate constant (k) and CO_2 concentration. Conversion rate of olivine as a function of dissolution rate constant k. (D) Conversion time of olivine as a function of dissolution rate constant k. The red and black squares represent the conversion times predicted by the D–P and combined SR + D–P mechanisms, respectively. The latter overlaps with the experimental conversion time of 2 h (Exp.). (E) The relationship between apparent dissolution rate constant k and CO_2 concentration. The gray area indicated the apparent dissolution rate change by ± 3 kJ/mol (from 34.0 kJ/mol to 40.0 kJ/mol). The figure shows that dissolution-precipitation mechanism alone cannot reproduce the experiment. By considering the CO_2 surface reaction, the apparent dissolution rate increases with log k' increasing from -7.11 to –4.08, and the estimated conversion time is significantly reduced. This reproduces experimental order of magnitude well, thereby helping to resolve the long-standing mismatch with experiments.

We have added a discussion of the remaining uncertainties associated with pH conditions in the "Surface carbonation facilitates cation dissolution" section as follows:

It should be noted that the carbonation-induced reduction in the Ca dissolution barrier under a

neutral condition and applied in the next section for neutral or close to neutral conditions, while future simulations with controlled protonation states would be useful for resolving the additional influence of local interfacial pH.

We have added a discussion of the remaining uncertainties associated with water conditions in the "Discussion" section as follows:

The role of CO_2 surface reactions may be underestimated if they are considered only under very water-limited conditions. Under extremely low water content, CO_2 surface reactions may occur on mineral surfaces, but the subsequent dissolution and precipitation processes are limited. As a result, their contribution to measurable carbonation conversion can be difficult to detect experimentally and considered as minimal. In contrast, we propose that under wet or partially wet conditions, CO_2 surface reactions can proceed continuously together with the dissolution–precipitation process. By reducing the dissolution barrier and enhancing the apparent dissolution rate as a function of CO_2 concentration, surface carbonation can effectively accelerate cation release, indicating that CO_2 surface reactions serve as an important additional mechanism for rapid carbon mineralization. The local water condition in the referenced experiments was not strictly controlled throughout the reaction, because water was added when the material appeared dry. Therefore, the system may have transitioned between aqueous and water-film conditions (< 0.7 g/g) [31] during carbonation. This introduces uncertainty into the prediction of carbonation conversion time, because water-film thickness can influence metal ion dissolution and the carbonation-induced reduction in dissolution free energy. Such sensitivity is consistent with previous demonstrations that pathway-based ion-release free energies at solid–water interfaces depend on the definition of the solution state and the configurational volume sampled by the detached ion [23]. Future studies combining controlled water/material-ratio experiments and simulations will be needed to quantitatively connect water-film thickness, dissolution energetics, and olivine carbonation kinetics.

We have added a discussion of the remaining uncertainties when considering in-situ carbon mineralization in the "Discussion" section as follows:

The reaction mechanism identified for Ca-olivine CDR materials is remarkably consistent with that observed in basaltic carbon mineralization systems. Our result shows that both surface Ca^{2+} and inner Ca^{2+} facilitate the reaction of CO_2 with NBOs in olivine. It implies that Mg-rich basalt reservoirs could also benefit from trace amounts of Ca^{2+} through a disproportionate amplification of carbonation kinetics. Although Ca^{2+} constitutes only 0–1 wt% of igneous olivine,[12] its reactivity exceeds that of Mg^{2+} by up to three orders of magnitude (log k'). Moreover, Ca is commonly enriched at the rims,[32] indicating that it may play a key role in initial dissolution and early-stage mineralization. Olivine can also benefit from additional Ca^{2+} released by coexisting minerals such as pyroxene and plagioclase. The adsorption of this dissolved Ca^{2+} onto olivine surfaces may further enhance its reactivity relative to pure Mg-olivine. Fe is another major cation in natural olivine. In this study, however, we focus first on Ca and Mg to isolate the effect of interfacial carbonation on dissolution kinetics. This simplification is also consistent with the experimental system used for validation, in which Fe was not explicitly considered. Quantifying the influence of Fe on interfacial carbonation pathways and dissolution kinetics therefore represents an important direction for future work. It should be noted that natural basaltic systems are considerably more complex than the simplified systems considered here. They commonly contain mixed mineral assemblages, Fe-bearing olivine, Al-bearing phases, impurities, heterogeneous surfaces, variable reactive surface areas, fluid-flow effects, and spatial variations in pH. This

study highlights the critical role of surface reactions in driving carbon mineralization, bridging the gap between slow carbonate precipitation kinetics and fast carbon mineralization field results [16]. The multiscale framework developed here offers a generalizable approach across multicomponent silicates, basaltic rocks, and industrial byproducts, offering guidance for scalable CDR strategies under diverse environmental settings. We hope that this study can be stimulating for further research in field applications.

2.1. Besides this general request, I have one main specific concern: Focusing on the theoretical methods used, the combination of ab initio molecular dynamics and metadynamics is an established and suitable choice for identifying and quantifying novel reaction pathways at surfaces. However, they remain limited in lengthscale, timescale, complexity, and fundamental accuracy. A key weakness of the present manuscript is the justification for the DFT simulation protocol – this presently uses PBE-D3 without justification. Either through finding appropriate references or additional computation, some evidence of the accuracy of this choice is needed. E.g. Reaction barriers are found to be the same as when higher-rung functionals are used. Approximations introduced at this stage could bias the observed reaction paths.

Response:

We thank the reviewer for this comment. We agree that the choice of exchange-correlation functional and dispersion correction should be more clearly justified. In the revised manuscript, we have expanded the Methods section to clarify the rationale by providing relevant references for using PBE-D3.

PBE provides a well-established description of oxide and silicate surfaces, while the D3 dispersion correction is needed to account for long-range van der Waals interactions that are important for CO_2, H_2O, and supercritical CO_2 at mineral interfaces. This approach has been widely used in ab initio molecular dynamics studies of CO_2–H_2O–mineral systems, including water/scCO_2/mineral interfaces, hydrated Ca-rich clay minerals, carbonation and hydrolysis on quartz and basalt surfaces, and mineralization simulations.

Several independent studies using related simulation protocol have reached consistent conclusions. For clarity, we have listed the relevant references below:

[1] Lee, Mal-Soon, et al. Structure, dynamics and stability of water/scCO_2/mineral interfaces from ab initio molecular dynamics simulations. *Scientific Reports* **2015**, *5* (1): 14857. (Ref. 36 in our manuscript)

> In this reference, using the Blue-Moon sampling method, the free energetics of the dissolution process by computing the potential of mean force (PMF) for Ca dissolution and CO_2 surface reaction. It has been observed that CO_2 surface reaction at NBOs formed by Ca-vacancy sites are barrierless. This is similar to the NEB results of this current work, with some difference in the local condition of NBOs.
>
> In their paper, the choice of DFT-D3 was justified by citing additional references (as follows):
>
> *"Dispersion corrections were accounted for by Grimme's third generation corrections*

DFT-D3[47] that have been evaluated extensively in the literature for both $scCO_2$ and H_2O and shown to adequately represent weak interactions in these systems[16,17,38,48,49,50]."

[16] Yoon, Y. et al. First-Principles Study of Phenol Hydrogenation on Pt and Ni Catalysts in Aqueous Phase. *Journal of the American Chemical Society* **2014**, *136*: 10287–10298.
[17] Lee, M.-S., McGrail, B. P. & Glezakou, V.-A. Microstructural Response of Variably Hydrated Ca-Rich Montmorillonite to Supercritical CO_2. *Environmental Science & Technology* **2014**, *48*: 8612–8619.
[38] Schaef, H. T. et al. Surface Condensation of CO_2 onto Kaolinite. *Environmental Science & Technology Letters* **2013**, *1*: 142–145.
[48] Balasubramanian, S., Kohlmeyer, A. & Klein, M. L. Ab Initio Molecular Dynamics Study of Supercritical Carbon Dioxide Including Dispersion Corrections. *Journal of Chemical Physics* **2009**, *131*: 144506.
[49] Grimme, S., Antony, J., Ehrlich, S. & Krieg, H. A Consistent and Accurate Ab Initio Parametrization of Density Functional Dispersion Correction (DFT-D) for the 94 Elements H-Pu. *Journal of Chemical Physics* **2010**, *132*: 154104.
[50] Windisch, C. F. Jr. et al. Raman Spectrum of Supercritical $C^{18}O_2$ and Re-Evaluation of the Fermi Resonance. *Physical Chemistry Chemical Physics* **2012**, *14*: 2560–2566.

[2] Li, Yunjian, et al. Ab initio mechanism revealing for tricalcium silicate dissolution. Nature Communications **2022**, *13* (1): 1253. (Ref. 39 in our manuscript)

This work also conducts ab initio metadynamics simulations to explore the mechanism of tricalcium silicate (Ca_3SiO_5) dissolution. It is mentioned:
All the AIMD simulations reported in this work were performed within the framework of DFT with the generalized gradient approximation (GGA) using the Perdew-Burke-Ernzerhof (PBE)[41] functional and Grimme D3 correction[42], which was implemented in the CP2K/Quickstep code[43].

[3] Chen Hsieh. Replacement reactions for carbon geosequestration may be faster in calcium olivine vs magnesium olivine. *Communications Earth & Environment* **2023**, *4* (1): 359. (Ref. 22 in our manuscript)

In this work, metadynamics were employed to compare the free energy barrier of Ca and Mg olivine using PBE-D3. It has been observed from simulation results that there is a 10^3 times dissolution rate constant difference based on energy difference, which is the level as reported in references (Palandri, J. L. & Kharaka, Y. K. A Compilation of Rate Parameters of Water-Mineral Interaction Kinetics for Application to Geochemical Modeling. Open-File Report; Wang, J. Thermodynamic equilibrium and kinetic fundamentals of oxide dissolution in aqueous solution. *Journal of Materials Research* **2020**, *35*, 898–921.).

[4] Shao Zihua, et al. Surface Reaction of CO_2 with Basaltic Minerals as a Mechanism for Carbon Mineralization. *Environmental Science & Technology* **2025**, *59* (28): 14410-14418. (Ref. 17 in our manuscript)

This is our previous work using the same DFT settings. Recently experiments in ref (Andrea Conti, et al. Molecular Views of Mineral Carbonation: Reaction of CO_2 with the

Wollastonite (100) Surface. *ACS Nano* **2026**, *20* (13): 10456-10465.) found experimental evidence of the surface reaction of CO_2 on wollastonite ($CaSiO_3$) surface, a similar surface as Ca-olivine (Ca_2SiO_4).

In their work, they also used a higher-rung meta-GGA functional, r^2SCAN+rVV10, which provides an improved description of both solid-state properties and nonlocal dispersion interactions. It was found that the reaction of NBOs with $CaSiO_3$ is also almost barrierless, like the NEB results of this current work. The remaining differences can be from the structural difference between $CaSiO_3$ and Ca_2SiO_4.

[5] Jia, Jihui, et al. Ab initio molecular dynamics study of carbonation and hydrolysis reactions on cleaved quartz (001) surface. *The Journal of Physical Chemistry C* **2019**, *123* (8): 4938-4948. (Ref. 38 in our manuscript)
[6] Liang, Yunfeng, et al. Modeling CO_2–water–mineral wettability and mineralization for carbon geosequestration." *Accounts of chemical research* **2017**, *50* (7): 1530-1540. (Ref. 37 in our manuscript)

Here are two additional works from our group exploring CO_2−water−mineral interactions using the DFT same settings for reference. In Jia, Jihui, et al., it is mentioned that:
"Grimme's third-generation correction DFT-D3 (51) was employed for dispersion corrections due to its superior performance for large-scale MD simulations based on the computation of forces and combination with standard functionals. (52) Intermolecular interactions play an important role when CO_2 molecules interact with the SiO_2 substrate. (39,40,53)".

[39] Hermann, Jan, and Ota Bludský. A novel correction scheme for DFT: A combined vdW-DF/CCSD (T) approach. *The Journal of Chemical Physics* **2013**, *139* (3): 034115.
[40] Malyi, Oleksandr I., et al. A first principles study of CO_2 adsorption on α-SiO_2 (001) surfaces. *Physical Chemistry Chemical Physics* **2015**, *17* (31): 20125-20133.
[53] Boström, Mathias, et al. Increased porosity turns desorption to adsorption for gas bubbles near water-SiO_2 interface. *Physical Review B* **2015**, *91* (7): 075403.

Same method has been applied for a basalt andesine surface studying CO_2 reaction in Liang Yunfeng, et al.

Action:

We have added the details in the "DFT parameters" section as follows:

Dispersion corrections was accounted for using Grimme's third generation DFT-D3 correction [35], because long-range van der Waals interactions are important for describing CO_2 and H_2O adsorption at mineral interfaces, and PBE-D3 has been widely applied in simulations of CO_2–H_2O–mineral systems [17,22,36–39].

2.2. Additional minor concerns:

Overall the manuscript would benefit from additional editing to make the claims clearer and to include references to other pertinent literature:

2.2a. The abstract should be lightly edited e.g. line 19 "However, the origin of the drastic reactivity among olivine.." is incomplete and should state what is varying (?) drastically about the reactivity. Similarly, line 29 presumably refers to *previously* predicted rates.

Response:

We thank the reviewer for this helpful comment. The abstract has been revised to clarify that the "drastic reactivity" refers to the reactivity contrast between Ca- and Mg-bearing olivines, and that the comparison with predicted rates refers specifically to previously predicted carbonation rates.

Action:

We have modified the Abstract as follows.

Ultramafic rock represents a vast, global resource with a theoretical capacity sufficient to store all current and historical human CO_2 emissions through permanent carbon mineralization. Ca-olivine, synthesized via Ca^{2+}/Mg^{2+} exchange reactions, reduces the energy demand of direct air capture (DAC) by nearly half, while carbon mineralization with naturally occurring olivine in basaltic and ultramafic rocks remains the lowest-cost among current carbon dioxide removal (CDR) strategies. However, the origin of the drastic reactivity between Ca- and Mg- bearing olivine remains poorly understood, and dissolution–precipitation models alone cannot explain the rapid mineralization observed in field projects. By combining ab initio molecular dynamics, metadynamics, and geochemical modeling, we show that their reactivity differences arise not merely from the cation type as commonly assumed, but more fundamentally from how these cations modulate the activity of nonbridging oxygens (NBOs). During surface carbonation, NBOs act as the oxygen source for CO_2, and in Ca-olivine this process reduces the Ca^{2+} dissolution barrier by 60%, accelerating ion release by six orders of magnitude. The calculated apparent dissolution rate constants reproduce the experimentally observed complete conversion of Ca-olivine within hours under 1 atm CO_2, bridging the gap between previously predicted and observed carbonation rates. This multiscale framework reveals how surface reactions govern silicate carbonation and provides a predictive basis for modeling and engineering large-scale CO_2 mineralization in both synthetic materials and basaltic reservoirs.

2.2b. The first paragraph should contain additional citations to the recent literature to improve accessibility to non-specialists, e.g. line 40, "With global reserves of Mg-rich silicates exceeding 10^5 gigatons and annual mine tailings over 400 million tons, this material offers vast and scalable potential for permanent CDR." is unreferenced despite the plentiful literature.

Response:

We thank the reviewer for this valuable comment. We agree that citations are needed to improve the accessibility of the Introduction for non-specialist readers. We have therefore added relevant recent references to support the statements regarding the global CO_2 removal potential of Mg-rich silicates and the annual production of mine tailings.

Action:

We have added the relevant references in the "Introduction" section as follows:

Global reserves of Mg-rich silicates have the potential to remove 10^5-10^8 gigatons (Gt) CO_2 [2] and annual production of mine tailings about 420 million tons [3], this material offers vast and scalable potential for permanent CDR.

References:

2. Kelemen, P., Benson, S. M., Pilorgé, H., Psarras, P. & Wilcox, J. An Overview of the Status and Challenges of CO_2 Storage in Minerals and Geological Formations. *Front. Clim.* **1**, 9 (2019).
3. Kelemen, P. B. *et al.* Engineered carbon mineralization in ultramafic rocks for CO_2 removal from air: Review and new insights. *Chem. Geol.* **550**, 119628 (2020).

2.2c. There are various points throughout where the authors could be more precise and include references to prior work. E.g. line 67 "This suggests the presence of alternative carbonation mechanisms not captured by current models." (which?), line 72 "Although long considered kinetically negligible" (add reference).

Response:

We thank the reviewer for this helpful suggestion.

We have revised this paragraph to specify that the alternative mechanisms refer to direct CO_2–mineral surface reactions and surface complexation processes that are not explicitly represented in conventional dissolution–precipitation-based models.

We have also added a review article reference (Olajire, A. A. A review of mineral carbonation technology in sequestration of CO_2. J. Pet. Sci. Eng. 109, 364–392 (2013).) to support the statement "Although long considered kinetically negligible".

In the review paper, it summarizes:

"The direct reaction of gaseous CO_2 with solid mineral or alkaline waste is the most straightforward MCT route. As an example, the direct gas–solid reaction of solid Ca/Mg-silicate is given:

$$Ca/Mg - silicate_{(s)} + CO_{2(g)} \rightarrow (Ca/Mg)CO_{3(s)} + SiO_{2(s)} \quad (7)$$

However, it suffers from very slow reaction rates and has practically been abandoned. Only two research groups (Reddy et al., 2008, Baciocchi et al., 2009a, Prigiobbe et al., 2009d) are still somewhat involved in this process using reactive wastes, but this route is unlikely to develop further than the research stage and will not reduce CO_2 emissions substantially."

Action:

We have revised the relevant sentences in the "Introduction" section as follows:

This discrepancy suggests that additional carbonation pathways, particularly direct CO_2–mineral surface reactions and surface complexation processes, may contribute to mineralization but are not explicitly represented in conventional dissolution–precipitation-

based models. Direct gas–solid surface reactions are represented by reaction (1) on the mineral surface [14,17]:

$$Ca/Mg - silicate_{(s)} + CO_{2(g)} \rightarrow (Ca/Mg)CO_{3(s)} + SiO_{2(s)} \quad (1)$$

Although long considered kinetically negligible [14], here we propose that these surface reactions proceed with low activation barriers and can initiate carbonation processes that complement or even precede aqueous-phase pathways.

2.3. The figures are in general well-constructed, but a clearer overview figure of the surface geometry and proposed reactions considered is recommended – I do not find Fig 1F clear.

Other than the points noted above, the manuscript is in general clearly written.

Response:

We appreciate this valuable suggestion. We have revised Fig. 1 by moving the reaction equations for pathways 1 and 2 from the original Fig. 1F to the corresponding NEB calculation panels (Fig. 1C and D). The associated reaction configurations are also shown in Fig. 1C and D. In addition, Fig. 1F has been revised to illustrate the reaction pathway 1 and 2 more clearly.

Action:
We have modified Fig. 1F as follows:

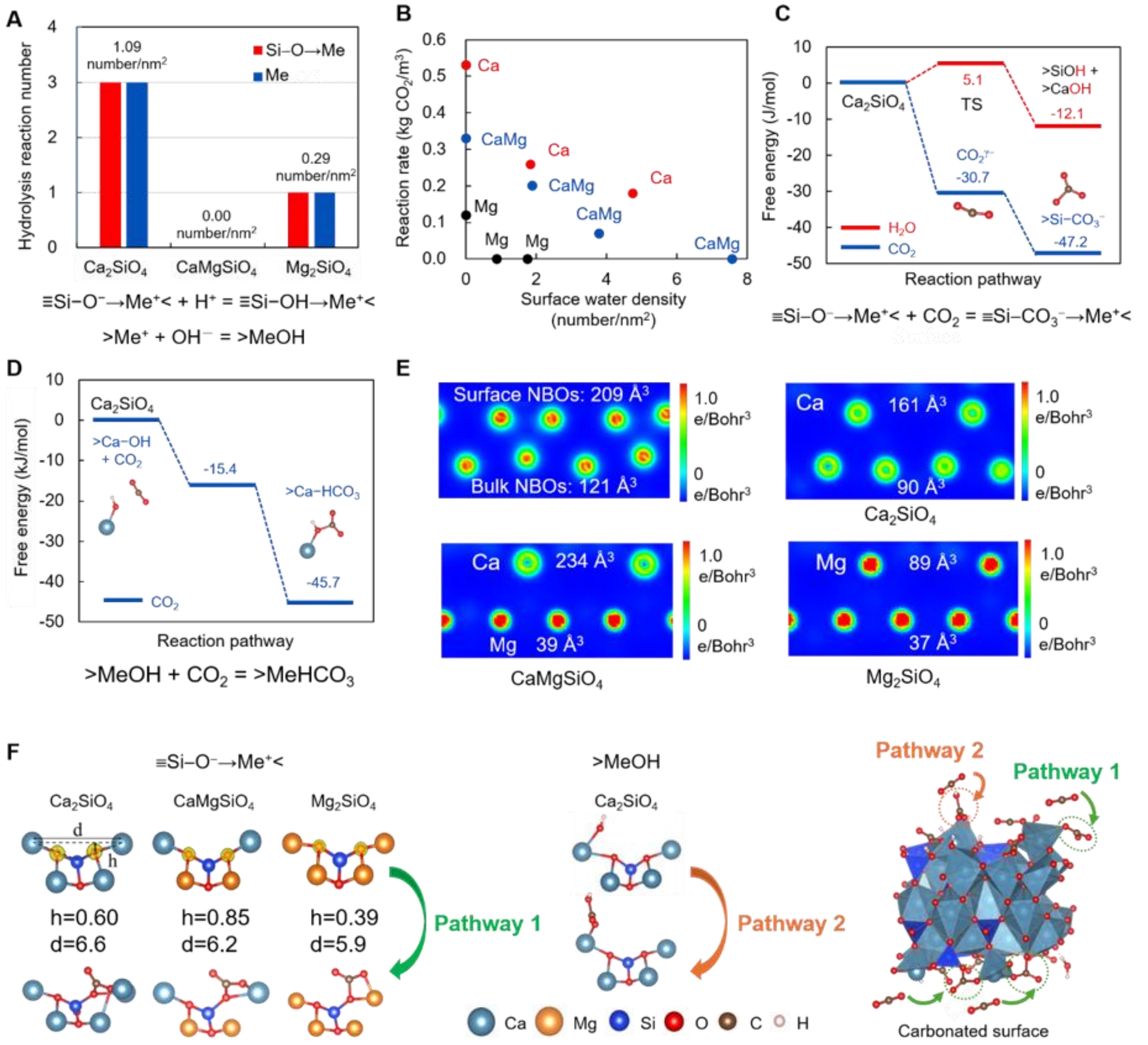

Fig. 1. Drastic reactivity difference between Ca and Mg in olivine. (A) Hydrolysis reactions on olivine surfaces and reacted water number per surface area. (B), CO_2 surface reaction rates under varying water coverage. (C) Binding energies of H_2O and CO_2 (pathway 1) on Ca_2SiO_4 surfaces. (D) Binding energy of CO_2 with Ca-OH groups (pathway 2). (E) Electron density distribution and atomic volumes of NBOs, Ca^{2+} and Mg^{2+}. (F) Schematic illustration of CO_2 surface structure on Ca-, CaMg-, and Mg-olivine before and after reaction. h and d in the unit of Å. The figure shows that the surface non-bridging oxygen (NBO) reacts more easily with CO_2 compared with water, and the surface reactivity with CO_2 follows an order: Ca-olivine > CaMg-olivine > Mg-olivine, stemmed from different ionic radius and surface geometries.

Reviewer: 3

Comments:

3.1. There are some key disconnects between this study and the natural system implications. I appreciate that the authors are simulating systems where mineral surfaces are in contact with water, as many molecular simulation studies react CO_2 with unrealistic mineral surfaces that will not be found at the surface or subsurface. However, it is not clear how these results will translates to aqueous environments, which are more abundant than the corresponding solid-water-gas surface or regolith environments.

Additionally, the authors are overstating the importance of Ca in olivine, which is only present in trace or minor amounts (<1%). The results about kinetic differences between how Ca- or Mg-rich olivine surface sites have not been shown by the authors to have a measurable impact in nature. Calculations or modeling could potentially be employed to strengthen the wider impact, but the small amount of Ca in olivine can't be overcome in terms of what is driving the reactions. If the energy barriers and dissolution kinetics for Mg are so much lower relative to Ca, Mg reactivity remains the controlling step of olivine reactivity overall. Overall this key disconnect limits the applicability and relevance to natural systems, but it is still interesting from a surface geochemistry perspective, just for a more specialist community.

I recommend this manuscripts be updated to more realistically portray the implications for rock-CO_2-water interactions, and then resubmitted to a more specialist journal focused on surface chemistry or broad mineralogy or geochemistry. A broader impact could be made if the authors also include the effects of iron, which is abundant (often 10-15%) as a cation substitute for Mg in olivine. One qualitative implication the authors may be able to suggest is that Ca may initially help olivine dissolution, as the <1% Ca in olivine tends to have a 10s-100s ppm enrichment in the rim compared to the core in natural olivine samples.

Response:

We thank the reviewer for this insightful and constructive comment. We fully agree that bridging simulations with natural system implications, particularly for in-situ carbon mineralization, is essential. We appreciate the opportunity to clarify and refine the scope of our conclusions.

Regarding the applicability to aqueous environments, we note that aqueous conditions were explicitly examined in the section "CO_2 uptake kinetics under ambient conditions" using PHREEQC. In this part, we focus on ex situ mineralization, namely aqueous carbonation under ambient air and 1 atm CO_2 conditions. This represents a one-dimensional carbonation prediction, and extension to in situ systems would require multiphase flow modeling beyond the scope of the present study. Regolith like environments may differ from fully aqueous systems in that mineral surfaces are often only partially wetted, allowing direct access of gaseous CO_2 to reactive surface sites through thin water films. In this context, our atomistic simulations are more representative of such partially wetted interfacial conditions than of bulk aqueous environments. Under these conditions, surface reactions can be understood as enhancing dissolution rates as a function of CO_2 concentration, or more specifically, the extent of CO_2 surface reactions.

It should be strengthened that the surface reaction between CO_2 and non-bridging oxygen will play an important role for Mg-olivine in the in-situ carbon mineralization as suggested by our recent work, Shao et al., *Environ. Sci. Technol.* **59**, 14410 (2025). Thus, the essence of the work is to show a much-reduced dissolution energy barrier of cations by carbonation. Although the dissolution kinetics for Mg are much lower relative to Ca, the activity of CO_2 is increased substantially under high-pressure reservoir conditions in comparison to the ambient pressure condition used in the current work, this can enhance the dissolution kinetics of Mg. Therefore, as mentioned in Discussion section in our manuscript (Line 377-382), "*Importantly, geochemical modeling over extended timescales highlights the critical role of surface reactions in driving carbon mineralization, bridging the gap between slow carbonate precipitation kinetics and fast carbon mineralization field results*[16]. *The multiscale framework developed here offers a generalizable approach across multicomponent silicates, basaltic rocks, and industrial byproducts, offering guidance for scalable CDR strategies under diverse environmental settings.*" More study is ongoing in our group, however, it is not trivial; to make the work mathematically sound, we must enable the surface reaction between CO_2-phase and mineral surfaces to be explicitly included in the geochemical modelling tool (that is, a tool also works in supercritical CO_2 environment).

We agree that Ca is present only in trace to minor amounts (<1%) in natural olivine, and that Mg may remain the dominant cation controlling bulk dissolution. We have therefore revised the manuscript to avoid overstatement and now emphasize that Ca may primarily contribute to the initial stages of dissolution, as it is often enriched at mineral rims in natural systems.

We also agree that Fe is an important component in natural olivine and may influence reactivity. However, Fe was not explicitly considered in the experimental system used for comparison (Chen Y. & Kanan M.W., *Nature* **638**, 972 (2025)). Therefore, this aspect is not considered in the present study. We have nevertheless added a discussion in the revised manuscript to highlight Fe as an important direction for future work, particularly in the context of in situ carbon mineralization.

Overall, we have revised the "Discussion" section in the manuscript to better position the findings especially in the context of natural systems and to more clearly define their scope and applicability to CO_2 mineralization processes.

Action:

We have expanded the discussion of the implications for in situ carbon mineralization in the "Discussion" section:

The reaction mechanism identified for Ca-olivine CDR materials is remarkably consistent with that observed in basaltic carbon mineralization systems. Our result shows that both surface Ca^{2+} and inner Ca^{2+} facilitate the reaction of CO_2 with NBOs in olivine. It implies that Mg-rich basalt reservoirs could also benefit from trace amounts of Ca^{2+} through a disproportionate amplification of carbonation kinetics. Although Ca^{2+} constitutes only 0–1 wt% of igneous olivine,[12] its reactivity exceeds that of Mg^{2+} by up to three orders of magnitude (log k'). Moreover, Ca is commonly enriched at the rims,[32] indicating that it may play a key role in initial dissolution and early-stage mineralization. Olivine can also benefit from additional Ca^{2+} released by coexisting minerals such as pyroxene and plagioclase. The adsorption of this dissolved Ca^{2+} onto olivine surfaces may further enhance its reactivity relative to pure Mg-olivine. Fe is another major cation in natural olivine. In this study,

however, we focus first on Ca and Mg to isolate the effect of interfacial carbonation on dissolution kinetics. This simplification is also consistent with the experimental system used for validation, in which Fe was not explicitly considered. Quantifying the influence of Fe on interfacial carbonation pathways and dissolution kinetics therefore represents an important direction for future work. It should be noted that natural basaltic systems are considerably more complex than the simplified systems considered here. They commonly contain mixed mineral assemblages, Fe-bearing olivine, Al-bearing phases, impurities, heterogeneous surfaces, variable reactive surface areas, fluid-flow effects, and spatial variations in pH. This study highlights the critical role of surface reactions in driving carbon mineralization, bridging the gap between slow carbonate precipitation kinetics and fast carbon mineralization field results [16]. The multiscale framework developed here offers a generalizable approach across multicomponent silicates, basaltic rocks, and industrial byproducts, offering guidance for scalable CDR strategies under diverse environmental settings. We hope that this study can be stimulating for further research in field applications.

Thanks a lot for the kind words and the consideration. We hope that our paper can be of merit for our community.

We shall look forward to hearing from you.

Yours sincerely,
Yunfeng Liang, the University of Tokyo

List of Responses to Reviewers' Comments and Suggestions

Dear Editor,

Thank you very much for your kind consideration and the reviewers' comments concerning our manuscript. We have carefully revised the manuscript to address the reviewers' concerns. Major revisions include:

1. To respond to **Reviewer 1**, we have added a reference in the discussion section.
2. To respond to **Reviewer 2**, we have added the simulation script of this work in the supplementary data.

Revisions made in response to the reviewers' comments are highlighted in blue in the revised manuscript, while revisions made in accordance with the checklist are highlighted in red. A clean version of the manuscript has also been submitted as instructed. Our point-by-point responses to the reviewers' comments, together with the corresponding revisions, are provided below.

Reviewer(s)' Comments to Author:

Reviewer: 1

Comments:

The revised version of the manuscript does addressed all reviewer concerns. Clearly the manuscript has gained in strength and clarity re both context and metadynamics/DFT technicalities. I would suggest the authors to explicitly cite the work of Xinping Zhu et al ["Molecular mechanisms of CO_2 mineralization on wetting nanoscale surfaces using molecular simulations and metadynamics" — Natcomm, 2025 doi: 10.1038/s41467-025-65794] in the main manuscript as an outlook of their work regarding the necessity to consider pH at a future step.

Response:

Thank you very much for this assessment and helpful suggestion. We have now cited the work of *Xinping Zhu et al.* in the Discussion section accordingly.

Action:

In the "Discussion" section:

The effect of pH was considered only in the geochemical modeling, whereas explicitly incorporating pH effects into molecular dynamics and metadynamics simulations [33] remains an important direction for future investigation.

Reviewer: 2

Comments:

In this resubmission the authors have done an excellent job of addressing the questions raised by all 3 prior referees. I (reviewer 2) previously requested justification of simulation protocols, figure improvements, and some improved referencing and phrasing. In their response the authors have justified the density functional PBE-D3 choice via citations to similar studies. The methods used are up to modern standards, and I find that the revised manuscript sufficiently clearly communicates its potential weaknesses. As also noted by reviewer 1 there are numerous factors (pH, water concentrations, composition, timescale) relevant in the real-world aqueous environment. In the revised manuscript the limitations of the study and focus on a particular simplified experimental situation are more clearly indicated. The figure 1 and several key points in the text have also been improved in this revised version, e.g. the previously ambiguous abstract.

From a simulation perspective, one remaining weakness concerns reproducibility – ideally all input files and output datasets would be provided. Although sufficient high-level details are given, a complete archive would allow other practitioners to e.g. verify details of the simulation supercell sizes and lattice constants used or repeat the calculations with higher fidelity methods in future. If it is the authors' intention to provide extensive files in the Supplemental Data, this is not clearly stated in the manuscript.

I recommend this article for publication. It provides interesting new insight into mineralization that I hope will spark follow-on research.

Response:

Thank you very much for your constructive comments on reproducibility. We have now included the simulation scripts/input files used in this study in the Supplementary Data. We have also revised the manuscript to clearly state that these files are provided as part of the Supplemental Data. We hope that this addition further improves the transparency and reproducibility of our computational work.

Reviewer: 3

Comments:

It is apparent that the authors responded to all reviewer comments, and have improved and tuned the implications and context. This timely manuscript on an important topic related to mineral surface reactivity is ready for publication.

Response:

Thank you very much for the assessment of our revised manuscript. We greatly appreciate your recognition of the improvements made in response to the reviewers' comments, as well as your supportive evaluation of the significance and timeliness of our work.

Thanks a lot for the kind words and the consideration. We hope that our paper can be of merit for our community.

We look forward to hearing from you.

Yours sincerely,
Yunfeng Liang, the University of Tokyo
